# The spectrum of breast *in situ* papillary carcinomas with invasion and invasive breast carcinomas with papillary features: an overview of histological subtypes and diagnostic challenges

**DOI:** 10.1111/his.70072

**Published:** 2025-12-17

**Authors:** Emad A Rakha, Puay Hoon Tan, Wendy A Raymond

**Affiliations:** ^1^ School of Medicine University of Nottingham Nottingham UK; ^2^ Pathology Department Hamad Medical Corporation Doha Qatar; ^3^ Luma Medical Centre, The Paragon Singapore; ^4^ Department of Surgical Pathology Flinders Medical Centre, Flinders University and Clinpath Pathology Adelaide SA Australia

**Keywords:** breast cancer, differential diagnosis, encapsulated papillary carcinoma, invasive papillary carcinoma, mucinous carcinoma, papillary DCIS, pathology, prognosis, solid papillary carcinoma

## Abstract

Invasive breast carcinomas with papillary features (IBCP) constitute a distinct and morphologically diverse group of breast cancers characterised by varying degrees of papillary architecture and invasive behaviour. IBCP encompass (1) papillary carcinoma *in situ* associated with invasion, (2) invasive solid papillary carcinoma (ISPC), (3) encapsulated papillary carcinoma (EPC)‐like invasive carcinoma, (4) Papillary DCIS‐like invasive carcinoma, (5) high‐grade carcinomas with EPC‐like or SPC‐like morphology, and (6) invasive papillary carcinoma not otherwise specified (IPC), including the tubulopapillary pattern. This review summarises the evolving classification, histopathological features, diagnostic criteria, differential diagnoses, clinical prognosis, and treatment implications of various subtypes of IBCP. Particular attention is given to the diagnostic challenges and clinical relevance of recognising these tumour types. Standardised diagnostic criteria and further research into the biological behaviour of these entities are essential to guide appropriate management and improve prognostication.

## Graphical abstract

Invasive breast carcinomas with papillary features (IBCP) comprise diverse subtypes with variable architecture, histopathology, and clinical behaviour. This review outlines evolving classification, diagnostic criteria, and treatment implications, highlighting challenges in distinguishing *in situ* from invasive lesions. Standardised diagnostic approaches are essential to improve consistency, prognostication, and patient management.

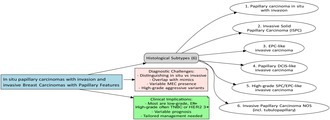

**Abbreviations**
AdCC: adenoid cystic carcinoma
EPC: encapsulated papillary carcinoma
IBC: invasive breast carcinoma
IBCP: invasive breast carcinomas with papillary features
IHC: immunohistochemical staining
ILC: invasive lobular carcinoma
IPC: invasive papillary carcinoma
ISPC: invasive solid papillary carcinoma
MECs: myoepithelial cells
NOS: not otherwise specified
SMMHC: smooth muscle myosin heavy chain
SPC: solid papillary carcinoma
TCCRP: tall cell carcinoma with reversed polarity
TDLU: terminal duct lobular unit

## Introduction

Invasive breast carcinomas with papillary features (IBCP), which account for less than 1% of breast cancers, represent a heterogeneous group of lesions that vary in clinical presentation, morphology, and biological behaviour, but share some common features: papillary architecture, an infiltrative growth pattern, and absence of myoepithelial cells (MECs).^1^, ^2^, ^3^, ^4^, ^5^, ^6^, ^7^, ^8^, ^9^ The criteria used to describe and classify these lesions in the literature vary considerably, resulting in some overlap and discrepancies.^3^, ^4^, ^10^ MEC markers are of limited utility in distinguishing carcinomas with encapsulated papillary carcinoma (EPC) or solid papillary carcinoma (SPC) growth patterns as *in situ* or invasive disease, as ~70%–80% of these tumours lack MECs but still behave as *in situ* lesions. As a result of the absence of MECs, a proportion of these tumours may be diagnosed in routine practice as invasive, thereby being erroneously grouped with more aggressive forms of conventional invasive breast carcinoma (IBC).

**Figure 1:**
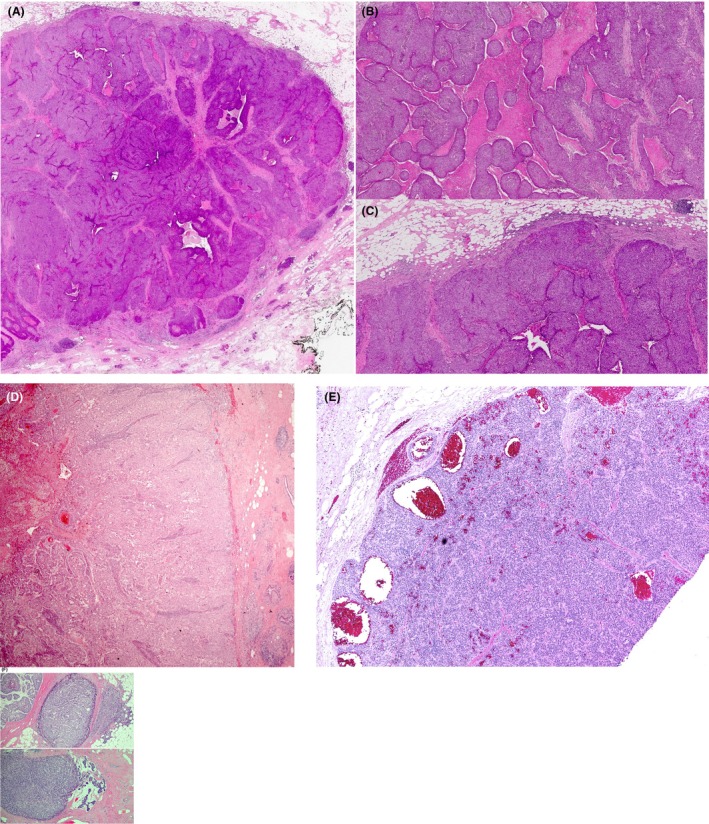
(**A**) An excision specimen of a case of IBC‐NST with a circumscribed SPC‐like architecture on low power (a), pseudopapillary (b), and entrapped stromal tissue (c) mimicking true cores of SPC. The tumour shows apparently defined (d) or focally irregular borders (a and c). (e) A case of triple‐negative high‐grade invasive breast carcinoma with entrapped stroma tissue mimicking papillary cores and defined borders mimicking SPC. This case was circulated in the UK external quality assurance (EQA) scheme, and 25% of the participants classified it as in situ carcinoma (SPC), whereas 75% classified it as invasive (SPC‐like) carcinoma.^10^ (f) A different case of invasive breast carcinoma with focal SPC‐like components, mucinous, and IBC‐NST components. This case is negative for MECs. (**B, C**) An excision specimen of a case of IBC‐NST with a circumscribed margin (a) and entrapped stromal tissue mimicking cores of SPC. The tumour shows a focally irregular margin (b), high‐grade features with high mitotic counts, increased nucleocytoplasmic features (c), and a triple‐negative phenotype (not shown).

Defining papillary architecture can also be challenging, making the application of specific diagnostic criteria subjective. Some tumours show classical papillary architecture with well‐developed fibrovascular cores covered by proliferating neoplastic epithelium and project into cystic spaces. In contrast, other papillary tumours exhibit a solid growth pattern with extensive proliferation of the neoplastic epithelial cell component, rendering the papillary cores subtle and rudimentary. Architectural patterns in breast carcinoma can be dynamic, and papillary morphology may even be observed in metastases from primary non‐papillary carcinomas, and vice versa.^2^, ^11^ Some high‐grade breast carcinomas with a high‐proliferative index may show tumour ischaemia, resulting in selective perivascular tumour cell survival and the emergence of a pseudopapillary architecture (Figure 1).^2^ Various other primary breast tumour types can exhibit papillary‐like architectural features, including secretory carcinoma, invasive lobular carcinoma (ILC),^12^ tall cell carcinoma with reversed polarity (TCCRP), mucinous cystadenocarcinoma,^9^ and adenoid cystic carcinoma,^9^ as well as metastatic tumours, such as serous carcinoma of gynaecological origin.

The broad spectrum of carcinomas with papillary architecture, overlapping histological features, and variable criteria for defining invasion has led to considerable variation in the reported frequencies of IBCP, ranging from 13% to 59% of malignant papillary lesions across different series^3^, ^4^, ^7^, ^9^ and from 0.002%^5^, ^6^, ^13^ to 0.03%^14^ of all IBCs.

Accurate diagnosis of IBCP is essential due to prognostic and treatment implications. Although the recognition of invasion in the context of EPC and SPC has increased, and is acknowledged in the 5th edition of the WHO classification of breast tumours,^9^ there remains a lack of comprehensive data on both the classification of the various forms of IBCP and the correlation between histological characteristics and clinical behaviour, as most earlier studies did not distinguish these tumour types. This review focuses on *in situ* papillary carcinomas with invasion and IBCs with papillary features, highlighting essential diagnostic features, differential diagnoses, diagnostic pitfalls, and management guidelines. It summarises the best available evidence and presents a pragmatic approach to assist in the identification and management of IBCP in routine diagnostic practice.

### Histological Entities of IBCP

There are multiple tumour types which can be regarded as IBCP. These tumours share a papillary architecture but show much variation in morphology and the nature of the papillary structures. Recognition of the diagnostic criteria is essential for accurate classification, which includes distinguishing between *in situ* and invasive disease and accurately typing the invasive carcinoma. IBCP include (1) Papillary carcinoma *in situ* (papillary DCIS, EPC or SPC) with invasion (the invasive component may or may not be papillary in nature), (2) Invasive solid papillary carcinoma (ISPC), (3) EPC‐like invasive carcinoma, (4) Papillary DCIS‐like invasive carcinoma, (5) High‐grade carcinoma with EPC‐like or SPC‐like morphology, and (6) invasive papillary carcinoma (IPC) not otherwise specified (NOS) (including the tubulopapillary pattern), in addition to IBCP that are mixed with other types of IBC (Table 1). It is also essential to exclude mimics, predominantly other special types of breast carcinomas with a papillary pattern, and metastatic tumours that exhibit a papillary architecture (see the section on differential diagnosis). IBCP are graded according to the Nottingham grading system.^15^ Tubule differentiation is usually absent and scored as 3, as papillary formation is not a feature of the normal breast and should not be considered as differentiation towards the structure of the normal terminal duct lobular unit (TDLU).

**Table 1:** Comparison of invasive papillary carcinoma subtypes of the breast

Subtype	Definition	Main features	Main differential diagnosis
1. Papillary carcinoma *in situ* associated with invasion	Papillary carcinoma *in situ* (papillary DCIS, EPC, or SPC) associated with a distinct invasive component (adjacent to the *in situ* lesion)	Invasive foci are most often IBC‐NST or mucinous carcinoma, occasionally papillary or other types. Stage, grade, and receptor assessment are derived from the invasive component	Distinguish from pure *in situ* carcinoma by IHC to demonstrate a lack of MECs coupled with stromal invasive features
2. Invasive Solid Papillary Carcinoma (ISPC)	Distinct invasive carcinoma subtype with solid papillary growth pattern and infiltrative jigsaw‐like architecture. Often shows neuroendocrine differentiation. If SPC progresses to mucinous carcinoma, the invasive tumour is classified as mucinous carcinoma	Often presents in older women. Has a favourable prognosis but metastatic potential. Shows absence of MECs, strong ER+, HER2‐ and are usually Grade 2	Differentiate from SPC *in situ* by the complexity of the architecture and stromal or fat infiltration. It should be distinguished from SPC with extracellular mucin or mucinous carcinoma
3. EPC‐like invasive carcinoma	Tumours with EPC‐like morphology but with multiple EPC‐like foci showing a complex architecture, irregular border, and focal invasive growth pattern	Complex cystic papillary clusters of variable size, with or without a dominant EPC‐like mass low grade, ER+, HER2−, and no capsule	Differentiate from classic EPC (circumscribed, encapsulated) by the complex architecture and from papillary DCIS by the prominent intracystic growth pattern and the larger size of the clusters. In some cases, it may be an index EPC‐like mass
4. Papillary DCIS‐like invasive carcinoma	Invasive carcinoma mimicking papillary DCIS, showing irregular borders and absence of MECs	Papillary clusters with fibrovascular cores and DCIS‐like appearance but infiltrative growth; absence of MECs confirmed by IHC	Differentiate from papillary DCIS (which has peripheral MECs) using IHC; maintain suspicion when features are atypical
5. High‐grade SPC or EPC‐like invasive carcinoma:			
5A. High‐grade SPC‐like invasive carcinoma	High‐grade tumours showing expansile, circumscribed margins with a solid papillary or pseudopapillary growth pattern	High‐nuclear grade pushing borders, but with at least focal stromal invasion that can be appreciated at high power magnification. Necrosis and frequent or atypical mitoses can be present. Often show a triple‐negative phenotype, and they all lack MECs	Differentiate from SPC *in situ* by the presence of high‐nuclear grade together with triple‐negative phenotype, focal stromal invasion or focal border irregularity. May show vascular invasion To be distinguished from ISPC by the circumscribed border and the high‐grade morphological features
5B. High‐grade EPC‐like invasive carcinoma	High‐grade EPC‐like tumours mimic EPC morphology but show focal invasive growth and high‐nuclear grade	High‐nuclear grade circumscribed or pushing borders, frequent stromal invasion, necrosis, and frequent mitoses. They are often triple‐negative or HER2‐positive	Differentiate from true EPC by high‐grade histological features, focal stromal invasion, or border irregularity. It may be associated with lymph node metastasis or vascular invasion and may coexist with other types of invasive carcinoma
6. Invasive Papillary Carcinoma, NOS (including tubulopapillary pattern)	Shows >90% papillary morphology, of infiltrative fibrovascular papillae in a desmoplastic stroma; includes tubulopapillary variant	Papillary fronds infiltrate a desmoplastic stroma, no capsule, variable grade. No surrounding MEC layer on IHC	Differentiate from micropapillary carcinoma (inside‐out growth pattern, no fibrovascular cores) and metastatic papillary carcinomas (serous carcinoma)

DCIS, ductal carcinoma in situ; EPC, encapsulated papillary carcinoma; SPC, solid papillary carcinoma; IBC‐NST, invasive breast carcinoma of no special type; MECs, myoepithelial cells; NOS, not otherwise specified.

IBCP are consistently characterised by the absence of MECs throughout, both at the periphery and at the stromal–epithelial interface of the papillary cores. IHC for MEC markers (such as p63, calponin and SMMHC) is a valuable adjunct in the diagnostic workup. However, it is essential to recognise that the absence of MECs alone is insufficient to confirm invasion in malignant papillary lesions. In EPC and SPC, a lack of MECs is frequently observed, despite the lesion having the clinical behaviour of carcinoma *in situ*. Therefore, the diagnosis of IBCP should only be made when the absence of MECs is accompanied by unequivocal evidence of stromal invasion, seen as irregular infiltrative margins, a desmoplastic stromal reaction, or entrapment of surrounding breast structures. Careful correlation with histomorphological features is essential to avoid overdiagnosis of invasion in papillary lesions that merely lack MECs.

Treatment of IBCP should be individualised, with complete surgical excision and clear margins in all cases, often supplemented by adjuvant radiotherapy in patients treated with breast‐conserving surgery. Sentinel lymph node biopsy is recommended. If there is any uncertainty regarding the invasive characteristics of a papillary tumour on a core biopsy, the pathology report should advise surgical excision to confirm invasion, and such tumours are not suitable for neoadjuvant therapy in the absence of definitive invasion. The outcomes of IBCP can be variable,^1^, ^4^, ^5^, ^6^, ^7^, ^8^, ^9^, ^13^, ^14^ and adjuvant systemic therapy may be warranted when the invasive component is extensive or exhibits adverse histological features. In general, most IBCP are low‐grade and strongly ER‐positive, and may be candidates for endocrine therapy, with minimal or no expected benefit from systemic chemotherapy.^1^, ^3^, ^4^, ^9^ However, when the invasive component is high grade or demonstrates an aggressive immunophenotype, such as triple‐negative or HER2‐positive, systemic chemotherapy or targeted therapies should be considered.^1^, ^3^, ^4^, ^9^

#### Papillary carcinoma *in situ* associated with invasion

**Figure 2:**
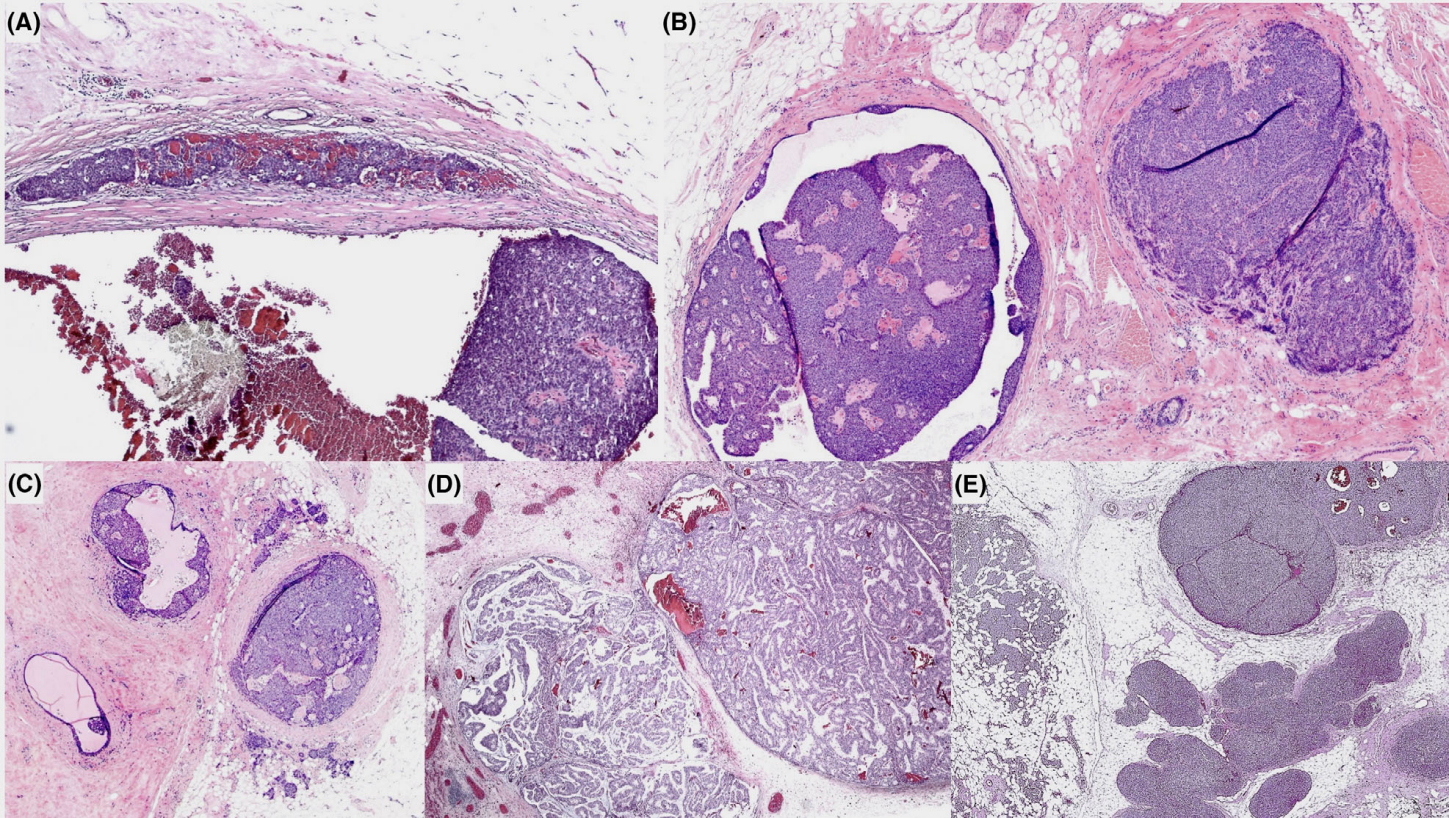
Different examples of papillary carcinoma in situ associated with separate areas of invasion. EPC (**A** and **D**), SPC (**B** and **E**), and papillary DCIS with invasion, respectively. In (A), the focus of invasion is more than 1 mm in size and appears distinct from the adjacent circumscribed cystic EPC, supporting early invasive disease. (B, D, and E) Two distinct foci of *in situ* and invasive disease, making it easier to measure the invasive tumour compared with the invasive foci in (C).

Papillary carcinoma *in situ* (classic EPC, SPC *in situ*, and papillary DCIS) can each be associated with invasive carcinoma, where a distinct, separate invasive component is identified adjacent to the *in situ* component (Figure 2). Histologically, the invasive component exhibits an infiltrative growth pattern characterised by irregular clusters, nests, tubules, single tumour cells, or tumour cells dispersed within pools of mucin. In contrast to papillary DCIS, which retains a peripheral layer of MECs at the epithelial–stromal interface of the outer contour of the affected duct, invasive foci show a complete absence of MECs. In SPC *in situ*, MECs are preserved around the *in situ* component in only 10–40% of cases, while they are consistently absent in the invasive component. In EPC, the MECs are also not identified around the conventional encapsulated *in situ* component in more than 80% of cases.^1^, ^16^, ^17^ When MECs are lacking in both components of the papillae and expanded duct contour, the invasive nature is inferred based on architectural features and the infiltrative growth pattern with stromal desmoplasia, contrasting with the circumscribed and well‐demarcated appearance of SPC *in situ* and the peripheral thick fibrous capsule in EPC.

The invasive areas may be an IBC of no special type (IBC‐NST), mucinous carcinoma, or other histological types. Mucinous carcinomas are more frequently seen in association with SPC than with EPC or papillary DCIS. Invasive foci are often of low to intermediate‐nuclear grade and cytologically resemble the adjacent *in situ* component. Accurate diagnosis requires careful microscopic examination of multiple sections to clearly demonstrate invasive growth with a stromal reaction and differentiate it from the expansile borders of *in situ* carcinoma. Immunohistochemical staining (IHC) can assist in confirming invasion, highlighting the absence of MECs. For prognostic and therapeutic purposes, assessment of grade, stage, and receptor status should be confined to the invasive component. However, whole tumour size should reflect the total extent, including both invasive and in situ elements, consistent with current practice for invasive carcinomas associated with DCIS, with a separate measurement of the invasive component.

**Figure 3:**
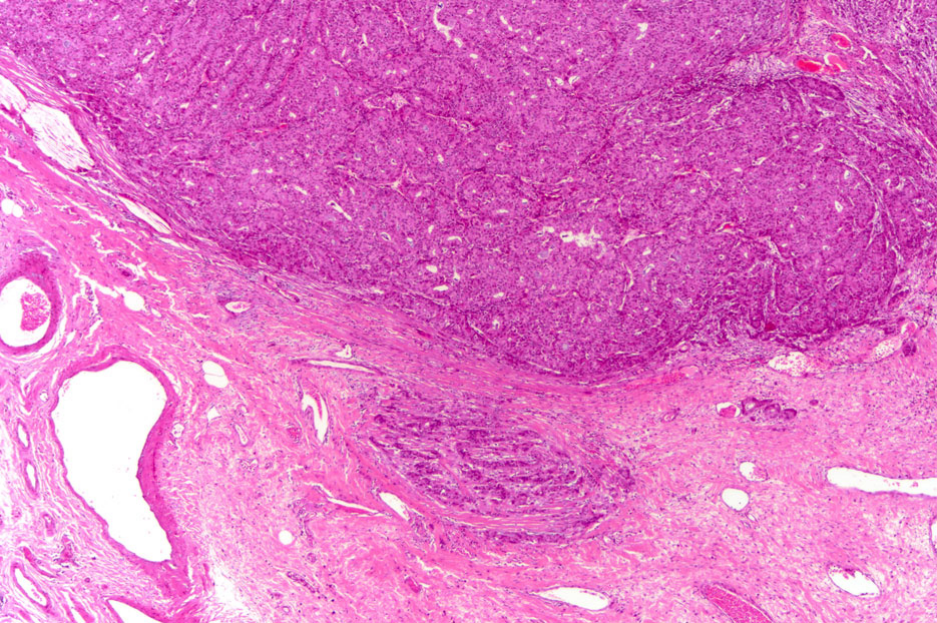
A case of SPC with focal stromal invasion less than 1 mm in size that is located outside the capsule‐like structure and lacks myoepithelial cells, so it is considered a microinvasion.

**Figure 4:**
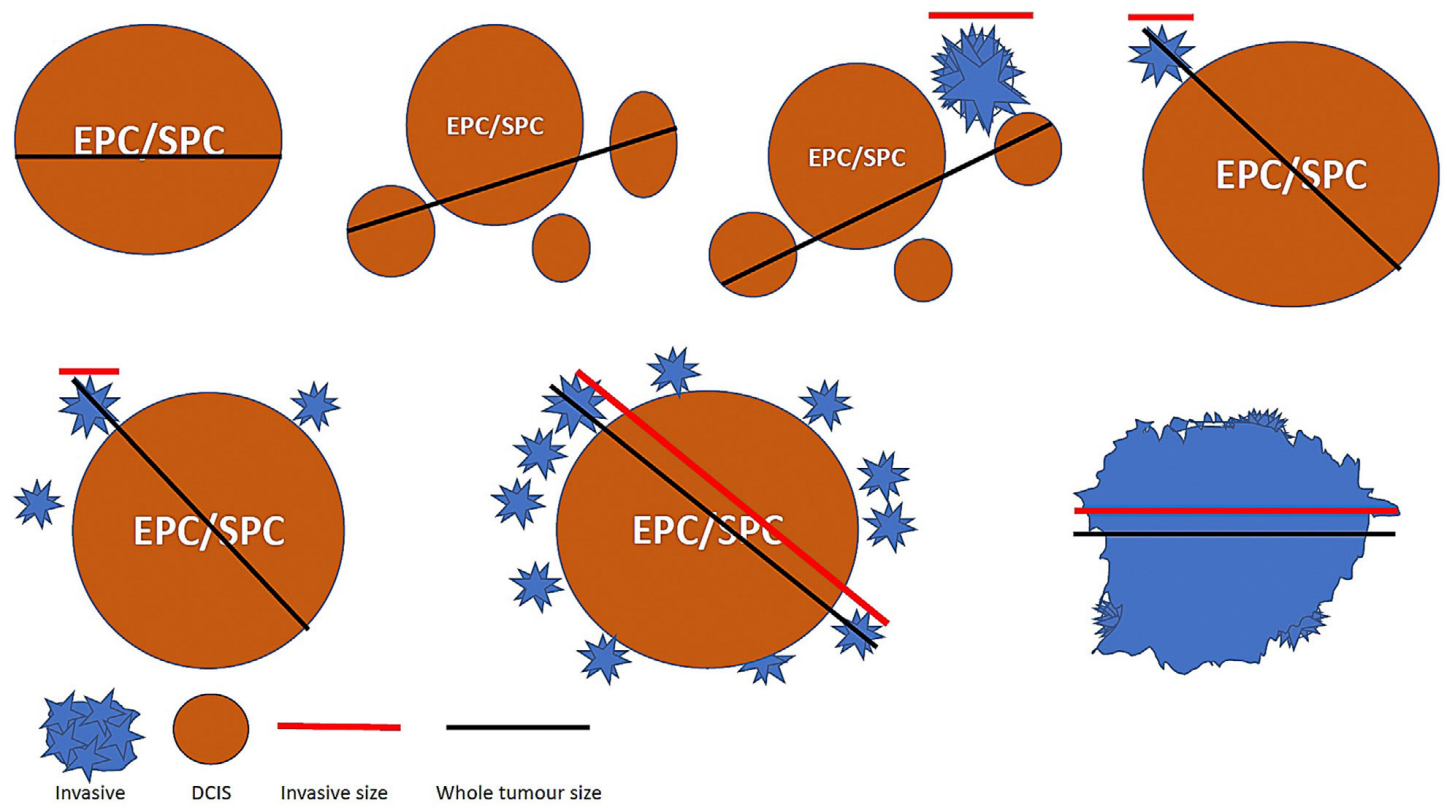
Schematic representation of the different scenarios of *in situ* (brown colour) and invasive components (blue colour) of solid (SPC) and encapsulated (EPC) papillary carcinomas when pure or mixed to demonstrate how to measure whole tumour size or DCIS size (black line) and invasive tumour size (red line) for management purposes.

Occasionally, EPC and SPC *in situ* exhibit an infiltrative border focally, with entrapped infiltrative‐appearing tumour foci within the surrounding fibrous tissue capsule or just adjacent to the main EPC/SPC tumour mass, mimicking conventional microinvasive foci (<1 mm in size) in the context of DCIS. The prognostic significance or the metastatic risk of these foci remain uncertain, given the recognised minimal invasive potential of EPC and SPC *in situ*.^1^, ^4^ Some of these foci may reflect biopsy site‐related tumour disruption with stromal reaction and entrapped tumour cells. IHC is often of little help as MECs can be absent in both *in situ* and infiltrative‐appearing components and may also be lost in the setting of core tract disruption. If true microinvasion is favoured (Figure 3), the tumour can be staged as pT1mi. Rarely, multiple foci of stromal invasion are seen at various sites around the nodules of EPC or SPC *in situ*, arguing against biopsy‐related effect, and the whole lesion can be considered invasive for tumour size measurement and TNM pT staging (Figure 4).

#### Invasive solid papillary carcinoma

Unlike EPC, the WHO working group classifies SPC into *in situ* or invasive.^9^ ISPC is a distinct histological subtype of IBC characterised by a solid papillary architecture and infiltrative growth pattern, with or without other features characteristic of SPC such as neuroendocrine differentiation and intracellular mucin accumulation.

Clinically, ISPC generally presents as a palpable breast mass in older women (≥ 50 years old), and its biological behaviour is considered favourable.^5^, ^7^, ^8^ While ISPC is often associated with an indolent course, there is documented metastatic potential, typically involving regional lymph nodes and occasionally distant sites.^6^ Consequently, accurate pathological diagnosis is crucial for clinical decision‐making.

Microscopically, ISPC lacks peripheral MEC layers and demonstrates an invasive growth pattern. Two main patterns of invasion are recognised in SPC.^9^

##### Infiltrative jigsaw‐like growth

**Figure 5:**
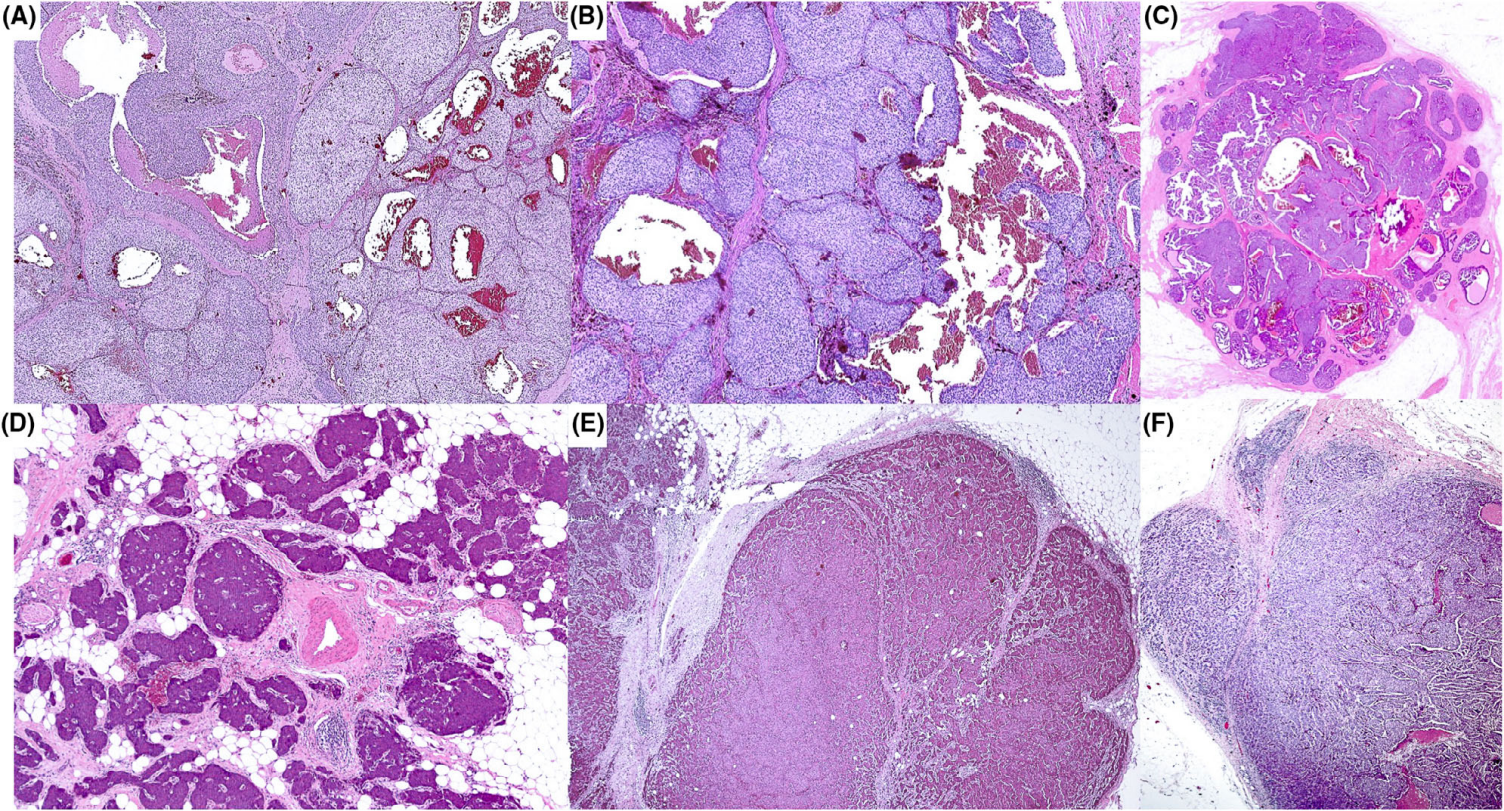
Examples of invasive SPC when the whole SPC tumour is invasive. Myoepithelial cells are absent in all these examples. In (**A**–**C**), the tumour forms a complex architecture with a jigsaw pattern, with little intervening stroma and no evidence of intraductal papillary growth. Focal stromal invasion by smaller clusters and single cells can be seen focally with a thorough examination. (**D**) shows large irregular solid papillary islands with infiltration of fat. Vascular invasion was seen in that case (not shown). (**E, F**) Tumours with focally circumscribed SPC‐like areas, but with marked fragmentation of the tumour and stromal invasion, featuring loose clusters of cells of variable size, mimicking IBC‐NST carcinoma.

**Figure 6:**
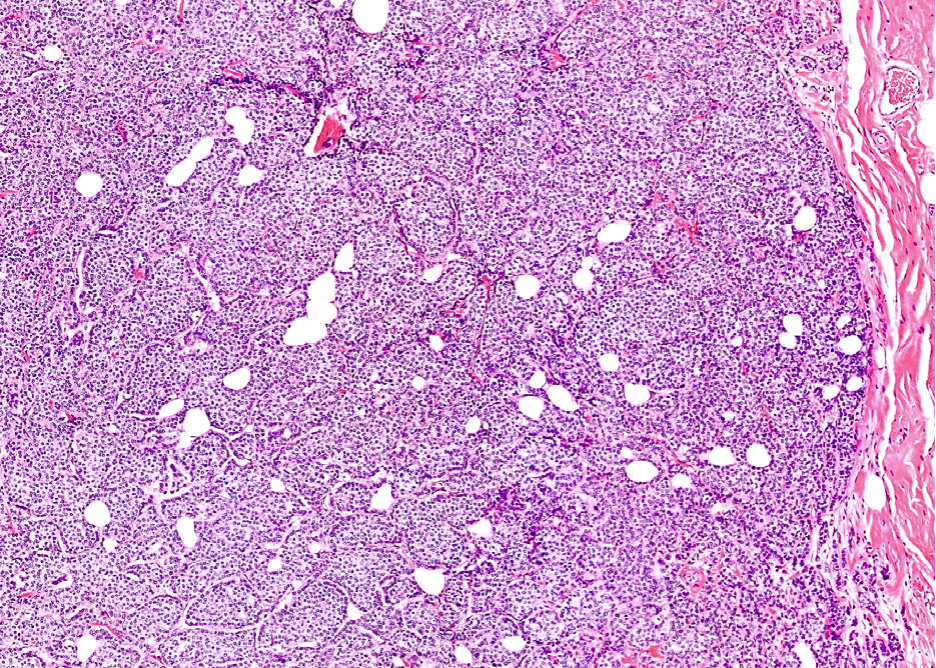
Fat infiltration is one of the signs of invasion in EPC and SPC. In this figure, a tumour mimics SPC, but fat cells are seen towards the periphery, confirming the invasive nature despite the apparently well‐defined pushing margin.

SPC *in situ* is characterised by well‐defined nodules and circumscribed masses showing solid papillary growth.^9^ These tumours usually lack a well‐developed peripheral fibrous tissue capsule, unlike EPC, and often exhibit a multilobated growth pattern. As SPCs *in situ* may lack MECs, the distinction between *in situ* and invasive lesions is based on the architecture. Invasion is suspected when the architecture is complex with multiple irregular clusters packed together, producing a geographic jigsaw‐like pattern (Figure 5). The current consensus classifies SPC with this jigsaw‐like pattern extending into the surrounding stroma and fat,^9^ or showing fat cells entrapped towards the periphery of the clusters (a sign of fat infiltration) (Figure 6),^4^ as invasive disease. These invasive foci retain a solid papillary architecture and are typically of variable size, but smaller than nodules of SPC *in situ*. Thorough examination often demonstrates areas with definite stromal invasion (e.g., single‐cell infiltration or IBC‐NST‐like foci) within the lesion (Figure 6). These ISPCs may exhibit high‐grade nuclear features, mitotic figures and lymphovascular invasion,^18^ and can morphologically overlap with so‐called “neuroendocrine tumours” of the breast.^19^ However, if the SPC architecture is evident, we recommend classifying such tumours with neuroendocrine features as ISPC rather than neuroendocrine tumour or carcinoma.^20^ SPC *in situ* also often shows neuroendocrine differentiation.

ISPCs are considered fully invasive carcinomas, although they generally predict a favourable prognosis.^5^, ^7^, ^8^ Some ISPCs show a biphasic growth pattern with a well‐circumscribed component and an infiltrative component (as defined by the complex jigsaw pattern or by fat infiltration) that maintains the SPC growth pattern. In such cases, IHC for MEC markers can be helpful to delineate the various components, but only if MECs are seen at the periphery of the *in situ* component. Therefore, it is our practice to perform IHC with MEC markers in such cases, as this can confirm the invasive nature in a proportion of cases and influence the assessment of invasive tumour size (through delineating the area for the evaluation of tumour grade and receptor status). We have encountered occasional SPCs with a somewhat complex architecture, suspicious of invasion, but IHC confirmed the preservation of the MEC layer and the *in situ* nature of the lesion.

##### Progression to hypercellular mucinous carcinoma

Intracellular mucin is frequently observed in SPC *in situ* and serves as a helpful feature in distinguishing it from EPC, together with other features including solid growth pattern, neuroendocrine differentiation, nuclear palisading, and streaming of spindled epithelial cells. While focal extracellular mucin may also be present within the lesion, the identification of abundant extracellular mucin or malignant epithelial cells floating within mucin pools should prompt consideration of mucinous carcinoma. In cases where extracellular mucin is extensive, together with floating tumour cells, the lesion should be classified as mucinous carcinoma associated with SPC.^21^, ^22^

**Figure 7:**
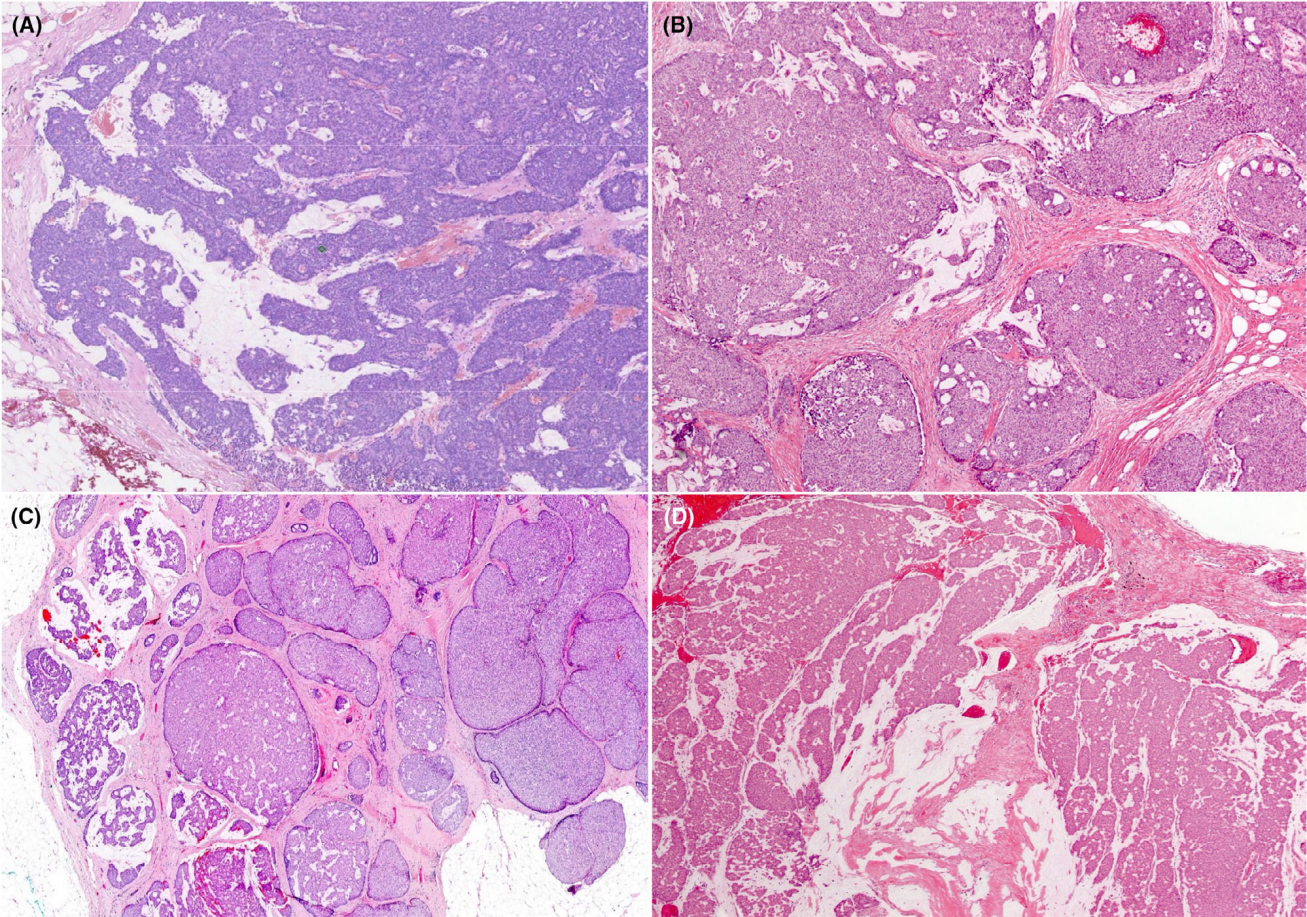
Various cases of SPC with increasing secretion of extracellular mucin, transforming the tumour to hypercellular mucinous carcinoma. Determining invasion is challenging in such early cases and is based on multiple features, including the amount of extracellular mucin or mucin lakes, floating malignant cells, and stromal infiltration in addition to the size of the area(s) showing such features. (**A**) and (**B**) can be considered microinvasion or early invasion based on the size of the affected area. Examination of the entire case and levels may help establish the diagnosis of mucinous carcinoma; otherwise, it can be considered a focus of microinvasion. Invasive carcinoma size in such a case can be limited to the areas with definite features of mucinous carcinoma, unlike the case in (**C**) and (**D**), which represent well‐established mucinous carcinoma. In (**C**), two components are present, *in situ* and invasive, whereas in (**D**), the whole lesion is a mucinous carcinoma.

**Figure 8:**
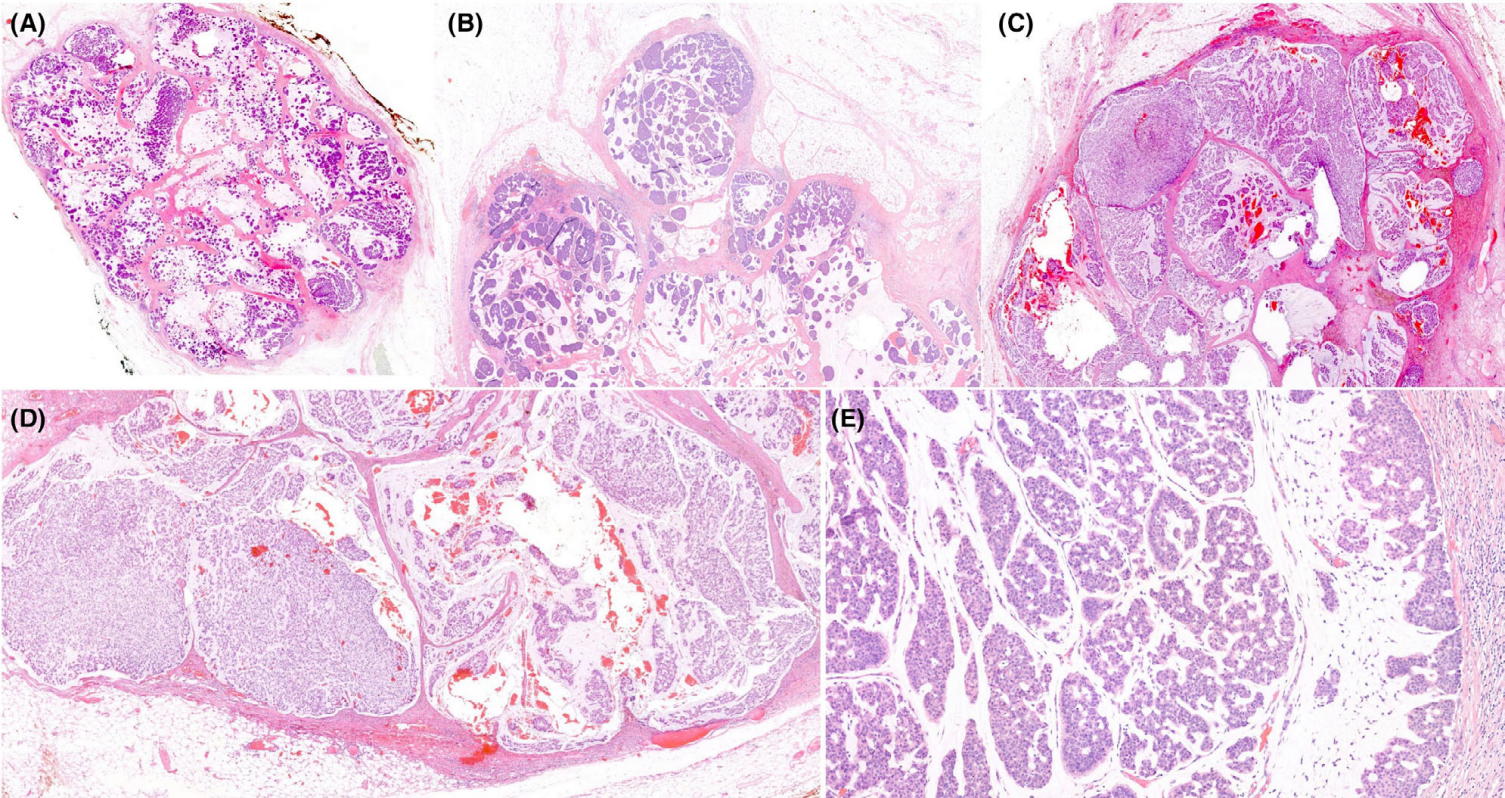
(**A**–**E**) Examples of SPC that progressed to mucinous carcinoma, and in such cases, the circumscribed borders, focal solid papillary architecture, and the neuroendocrine differentiation favour progression from SPC, while the amount of mucin lakes with floating tumour cells supports the diagnosis of mucinous carcinoma.

SPC progressing to hypercellular mucinous carcinoma represents a distinctive pattern of tumour progression, characterised by the transformation of SPC *in situ* into a mucin‐rich carcinoma, and is classified as hypercellular mucinous carcinoma rather than ISPC (Figures 7 and 8). Histologically, the tumour shows circumscribed mass(es) of SPC with stromal infiltration associated with mucin secretion. The transition is often gradual, with some cases displaying overlapping features, ranging from SPC with focal extracellular mucin at one end of the spectrum to tumours with features of classic mucinous carcinoma at the other.

The distinction between SPC *in situ* and SPC associated with microinvasive, or early invasive, mucinous carcinoma is often subjective and is based on the extent of extracellular mucin accumulation, the number and size of mucinous foci infiltrating the stroma at the periphery of the lesion, and the presence of malignant cells within mucin pools that would be sufficient on their own to be classified as mucinous carcinoma. If in doubt, it would be appropriate to classify the tumour as SPC associated with foci of early invasion /progression to mucinous carcinoma and stage as pT1mi, if the likely invasive component does not exceed 1 mm.

If the foci of mucinous carcinoma are well developed and definite stromal invasion can be confirmed with malignant cells dispersed in mucin pools, the case should be considered a mucinous carcinoma, and prognostic markers such as size, grade, and receptors should be assessed. The amount of mucin is variable and inversely correlates with the degree of cellularity of the tumour. Unlike classic (Type A) mucinous carcinoma, which typically exhibits sparse cellularity within extensive pools of mucin, hypercellular mucinous carcinoma (Type B) arising from SPC is characterised by tumour cells arranged in solid papillary clusters and large nests with neuroendocrine differentiation suspended within mucinous lakes. These tumours are typically of low grade and show a luminal phenotype, reflecting tumour aggressiveness between SPC *in situ* and IBC‐NST.^23^, ^24^

Immunohistochemical profiling of ISPC frequently reveals diffuse positivity for neuroendocrine markers, such as INSM1, chromogranin, and synaptophysin, along with strong ER positivity, HER2 negativity, and the absence of MEC markers, such as p63, smooth muscle myosin heavy chain (SMMHC), and calponin. Clinically, these tumours tend to present as mass lesions^25^ but at a less advanced stage compared with classic mucinous carcinomas and demonstrate lower rates of lymphovascular invasion and nodal metastasis.^23^, ^24^

### Other Considerations

Some IBC‐NST may mimic SPC if presenting with an overall circumscribed architecture, stromal entrapment, and delicate fibrovascular strands resembling papillary cores. However, these tumours often lack other features commonly associated with SPC, such as neuroendocrine differentiation, intracellular mucin, and nuclear palisading, and often demonstrate irregular borders or focal areas with typical IBC‐NST morphology. These tumours that do not fulfil the features of ISPC may be regarded as SPC‐like IBC‐NST or IBC‐NST. The prognostic significance of SPC‐like morphology within IBC‐NST remains uncertain.

In some cases of ISPC, the invasive tumour clusters lose their solid papillary architecture and show an infiltrative pattern like that of IBC‐NST. These foci should be classified as IBC‐NST, admixed with ISPC, and prognostic variables should be selectively assessed in these areas if they possess higher grade appearances, rather than on the ISPC component.

In rare cases, metastatic carcinoma with SPC‐like morphology is identified in axillary lymph nodes, but the invasive tumour in the breast shows IBC‐NST morphology. In such cases, the breast tumour is classified as IBC‐NST (with mention of the different differentiation pattern in the node, to highlight the possibility of a second tumour in the breast). The more likely possibility in such cases is the spatial and temporal dynamic process of differentiation of IBC with a c and non‐papillary morphology.^2^

#### EPC‐like invasive carcinoma

**Figure 9:**
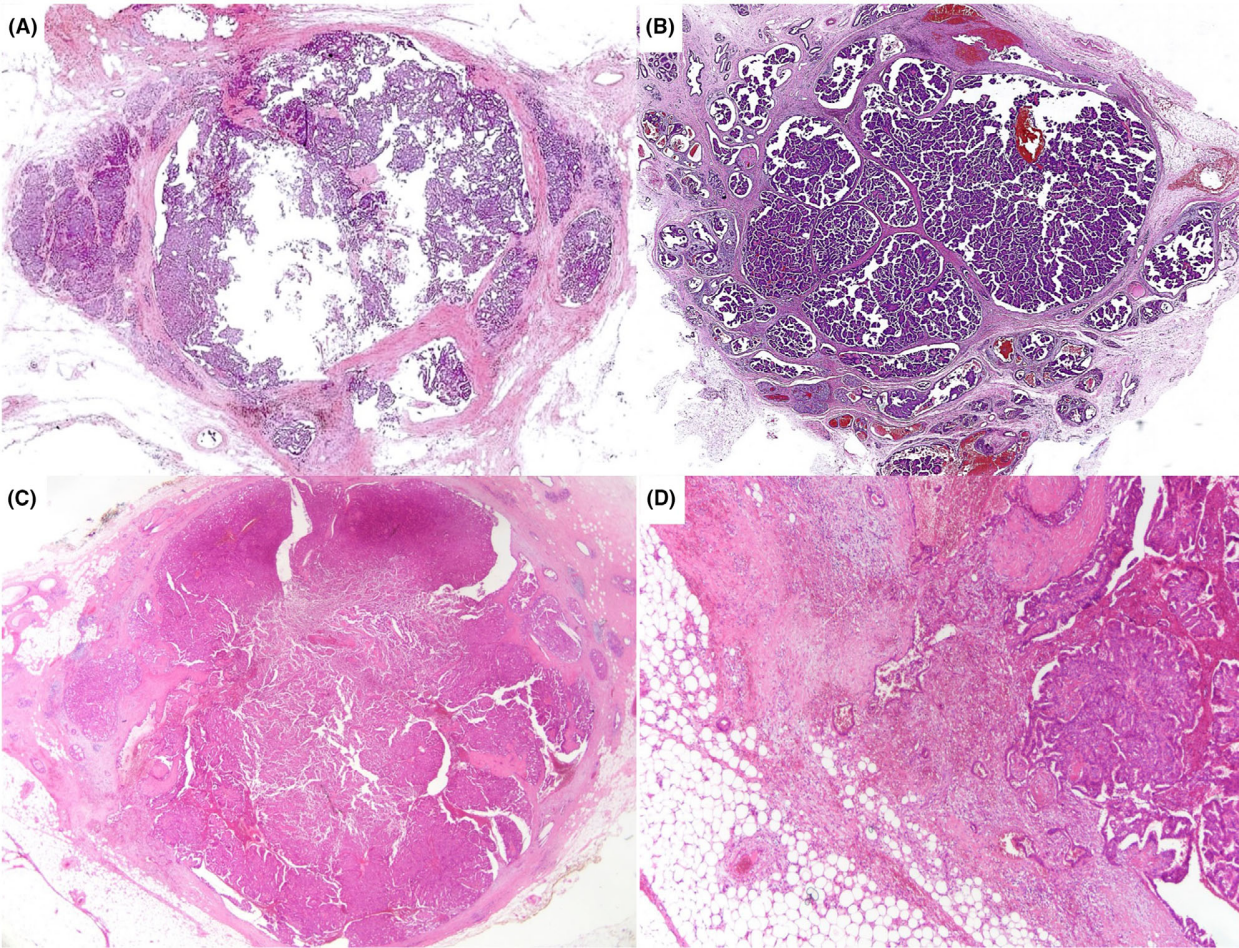
Three examples of invasive breast carcinoma mimicking EPC. (**A**) a central large cystic papillary mass mimicking EPC, but the border is irregular; it lacks a peripheral well‐developed capsule and is surrounded by multiple areas of stromal invasion alongside a lack of myoepithelial cells supporting the whole tumour as invasive disease. (**B**) Multiple irregular EPC with variable sizes and complex architecture. This, together with the lack of myoepithelial cells, favours the classification as an invasive tumour. (**C**) A case of circumscribed EPC‐like tumour with focal solid architecture and multiple areas of irregular borders and definite stromal invasion as shown in the high‐power view. (**D**) Favouring invasive tumour. Assessment of invasive tumour size in such examples is subjective and challenging, and a pragmatic approach is recommended, considering the most infiltrative areas of the tumour as invasive.

**Figure 10:**
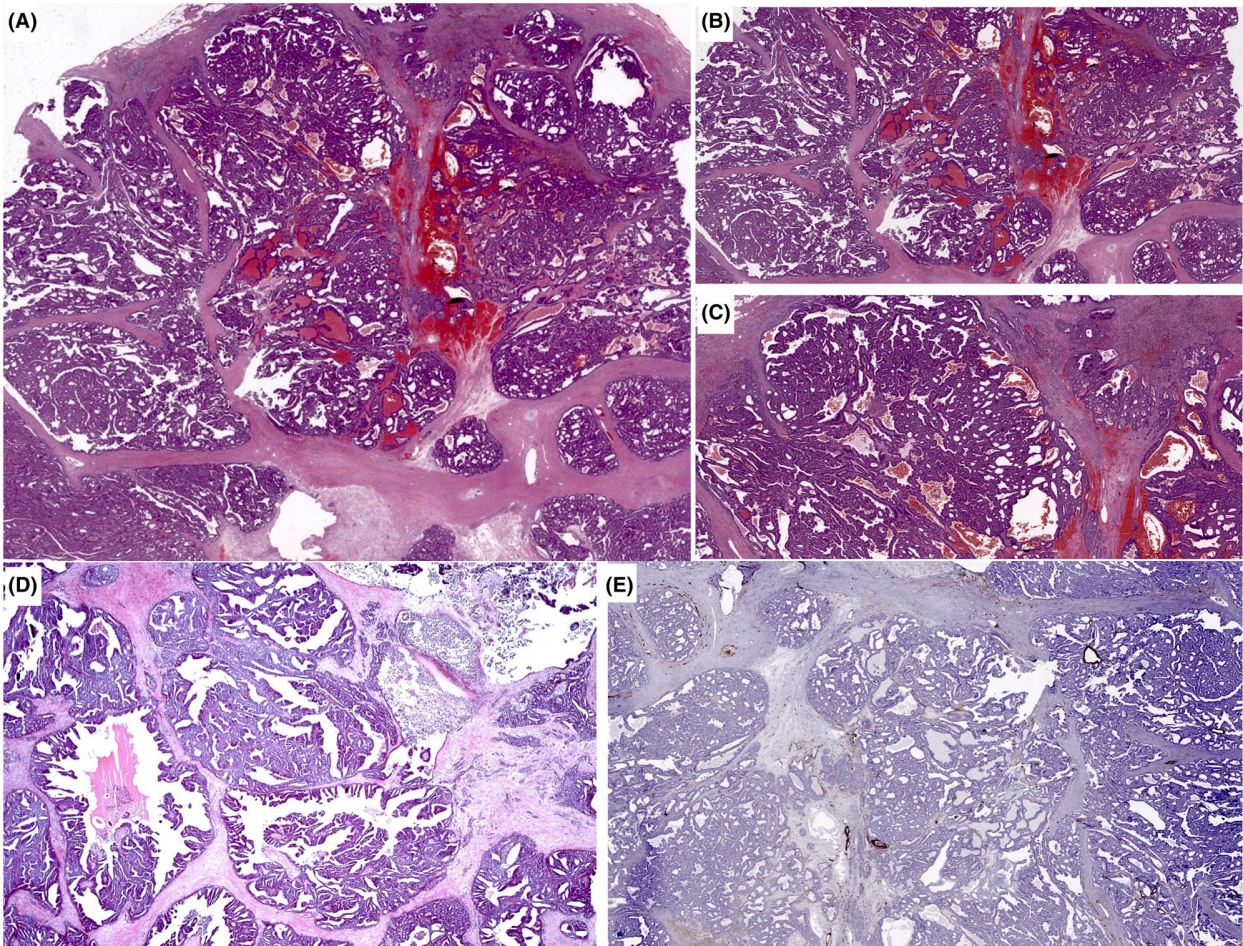
This tumour shows multiple EPC‐like nodules with irregular borders and complex architecture throughout the tumour, together with scattered foci of conventional‐type stroma invasion. (**A**–**D**) Variable areas at different magnifications. The lack of myoepithelial cells was confirmed using a panel of myoepithelial cell markers (SMMHC is shown in **E**).

Unlike SPC, which is classified as SPC *in situ* and invasive SPC (ISPC), the WHO working group describes EPC as a single entity, without adding the *in situ* designation or acknowledging a distinct invasive variant of EPC.^9^ However, tumours with EPC‐like morphology and an invasive growth pattern do exist. These lesions constitute a distinct subset of IBCP, characterised by invasive cystic papillary clusters exhibiting a complex architecture and irregular borders, together with a lack of MECs (Figures 9 and 10). There may also be focal or diffuse, more solid, irregular growth patterns. These clusters are typically larger than papillary DCIS, lack the solid growth pattern of SPC, and do not display the fibrous capsule or well‐demarcated margins of classical EPC. In our experience, such tumours show multiple complex cystic papillary clusters of variable size with (Figure 9) or without (Figure 10) a dominant EPC‐like mass. Immunohistochemical studies demonstrate a lack of MECs within and surrounding the areas of complex architecture, together with ER positivity and HER2 negativity. The classification of these tumours is subjective, and distinguishing *in situ* from invasive components can be particularly challenging. In such cases, we advocate measuring the invasive size based on areas with the most complex architecture, while the overall tumour size should reflect the entire lesion (Figure 4). This pragmatic approach acknowledges the uncertain biological behaviour of these tumours and avoids premature assignment to either end of the diagnostic spectrum when overlapping features are present.

#### Papillary DCIS‐Like Invasive Carcinoma

IBC mimicking papillary ductal carcinoma *in situ* (papillary DCIS) is a rare yet clinically critical diagnostic entity in breast pathology. These lesions pose substantial diagnostic challenges, as they closely recapitulate the architectural and cytological features characteristic of papillary DCIS, often resulting in the underdiagnosis of invasive disease.

The precursor lesion of such tumours remains undefined. Two plausible origins can be proposed: (1) They may arise from pre‐existing papillary DCIS, wherein multiple foci progress to invasive carcinoma via the loss of peripheral MECs and subsequent stromal infiltration; or (2) They may develop *de novo* (without a preceding papillary DCIS stage). In this latter scenario, the tumour acquires the morphological appearance of papillary DCIS, supported by two key observations: the absence of MECs surrounding all papillary DCIS‐like cell clusters, and at least some foci with the characteristic low‐power architectural morphology of papillary DCIS.

**Figure 11:**
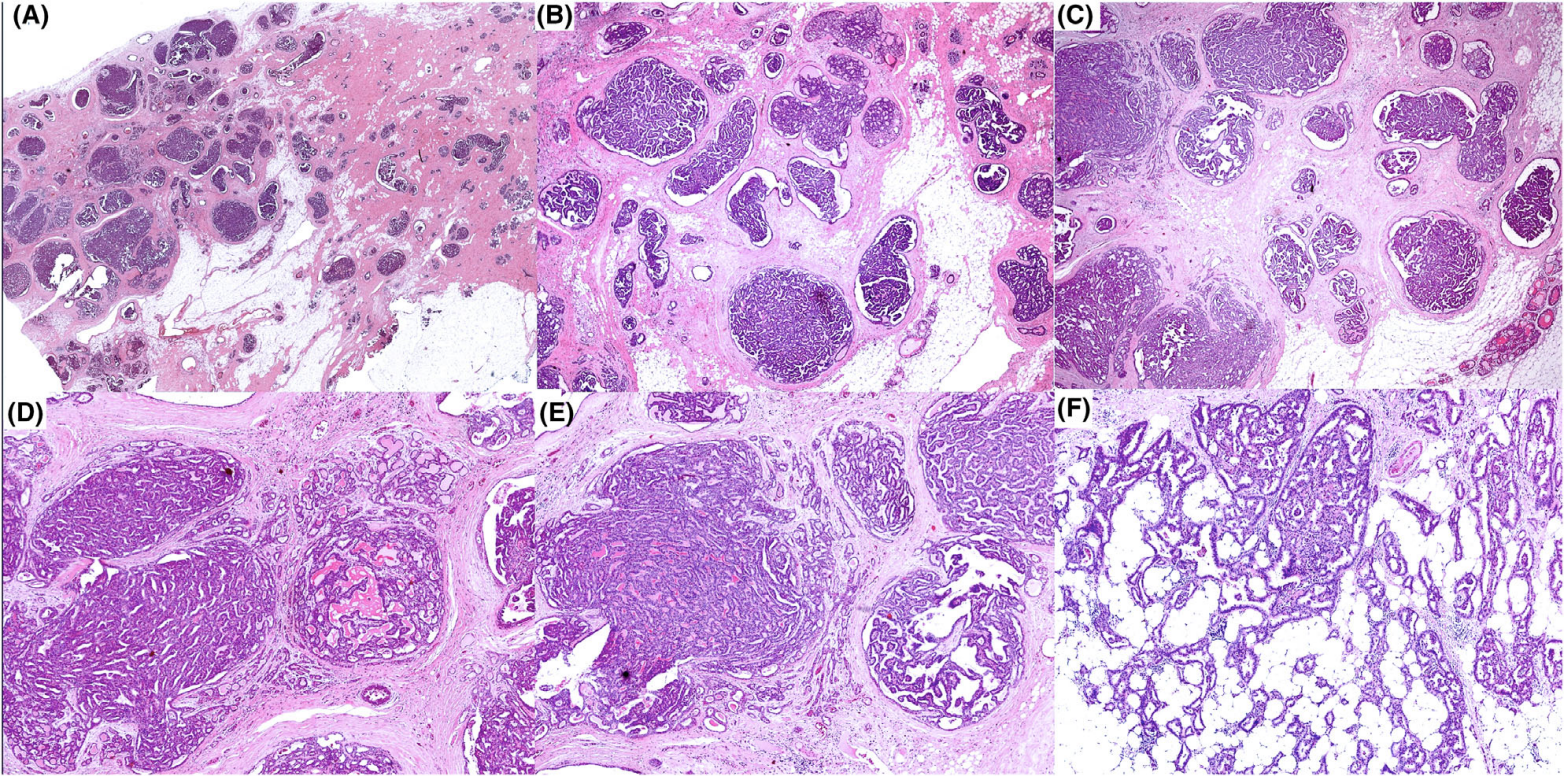
Papillary DCIS‐like invasive carcinoma can mimic papillary DCIS at low power (**A** and **B**), but examination at higher power and thorough evaluation of the borders of these variably sized papillary DCIS‐like clusters demonstrate irregular borders with multiple areas of definite stromal invasion (**C**–**E**) or even fat infiltration (**F**). The invasion was confirmed by the lack of a peripheral myoepithelial cell layer (not shown).

Microscopically, they demonstrate small‐ to medium‐sized clusters of well‐formed papillary structures with delicate fibrovascular cores lined by one to several layers of atypical epithelial cells closely resembling papillary DCIS on low‐power examination. The papillary clusters are of variable size, but typically smaller than seen in EPC and EPC‐like invasive carcinoma and show irregular infiltrative borders (Figure 11). The critical histological features that aid in recognising invasion in these lesions include irregularity of the borders of the papillary clusters, with multiple scattered foci of stromal invasion characterised by isolated or small groups of tumour cells permeating the adjacent stroma. Most importantly, the absence of the peripheral MEC layer at all tumour‐stromal interfaces, as confirmed by IHC, contrasts with that of papillary DCIS. Although these carcinomas typically demonstrate indolent biological behaviour, misdiagnosis as DCIS alone can lead to suboptimal treatment. Therefore, pathologists must maintain a high index of suspicion and apply meticulous histological and IHC assessment in cases of papillary DCIS, especially those with atypical features. A low threshold for employing MEC markers is recommended. Accurate diagnosis is essential for guiding appropriate surgical management, adjuvant therapy decisions, and patient follow‐up.

#### High‐Grade Carcinoma with EPC‐Like or SPC‐Like Morphology

Papillary DCIS, EPC, and SPC with a preserved peripheral MEC layer are classified as *in situ* carcinoma, regardless of nuclear grade or receptor status. Similarly, EPC and SPC lacking MECs but exhibiting classical phenotypic features (ER positivity, low to intermediate‐nuclear grade, and circumscribed borders with or without a fibrous capsule) are also classified as *in situ* carcinoma (TNM pTis) for management purposes.^9^ However, caution should be exercised before diagnosing high‐nuclear grade, circumscribed, solid, or cystic tumours lacking a peripheral MEC layer as *in situ* disease based solely on papillary architecture, as this may lead to undertreatment of an invasive high‐grade carcinoma.^10^, ^16^, ^26^, ^27^, ^28^, ^29^ Some high‐grade, rapidly proliferating IBCs exhibit a circumscribed or pushing border, and entrapped stromal tissue may mimic the papillary cores of SPC (Figure 1). Other tumours show an encysted architecture mimicking EPC but are of high nuclear grade and exhibit either a triple‐negative or HER2‐positive phenotype, with frequent or focal stromal invasion. These should be distinguished from typical EPC.^30^, ^31^, ^32^

Here, we propose that at least two distinct patterns exist: high‐grade SPC‐like invasive carcinoma and high‐grade EPC‐like carcinoma with intracystic architecture.

##### High‐grade SPC‐like invasive carcinoma

These tumours display pushing or circumscribed borders and a papillary or pseudopapillary architecture, often mimicking SPC (Figure 1). However, they are typically triple‐negative or HER2‐positive. Careful histological evaluation of the tumour margins often reveals subtle irregularities and unequivocal evidence of stromal invasion, distinguishing them from the intraductal growth patterns seen in SPC *in situ*. Despite their solid papillary‐like morphology, these tumours are likely to exhibit aggressive biological behaviour and should be classified and managed as IBC‐NST with SPC Pattern, with comprehensive grading, staging, and receptor profiling.

##### High‐grade EPC‐like carcinomas with intracystic architecture

**Figure 12:**
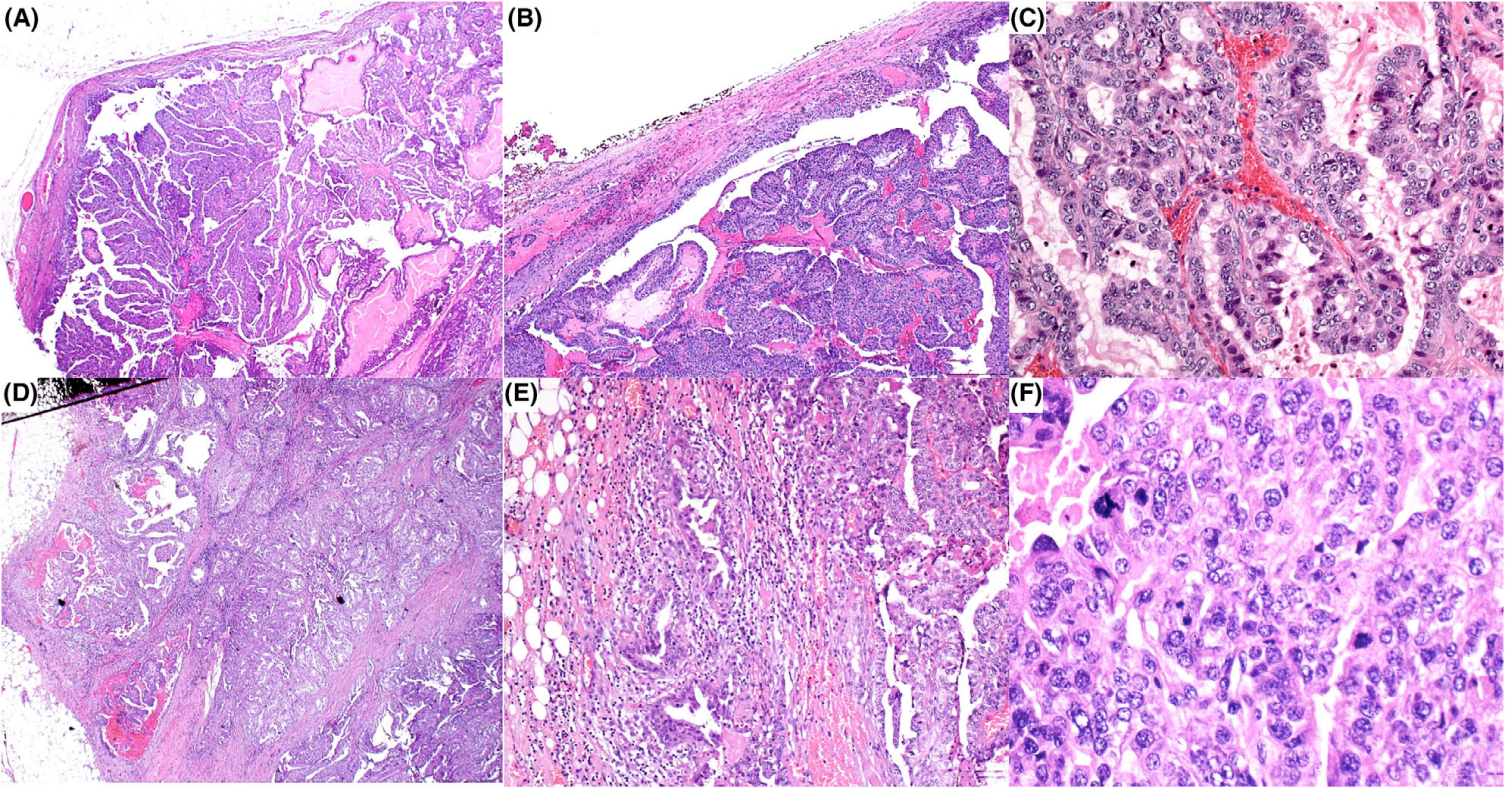
This case was presented in the core biopsy as part of high‐grade EPC, together with an ipsilateral lymph node biopsy that showed metastatic carcinoma with similar EPC‐like morphology and immunoprofile. It was difficult to classify the primary breast tumour as *in situ* (B5a), as it had a well‐defined margin and intracystic papillary growth pattern, or as invasive carcinoma (B5b), as it was high grade, showed triple‐negative phenotype, lacked myoepithelial cells, and was similar to that in the metastatic lymph node. On excision, the tumour showed focally well‐defined margins with capsule (**A** and **B**), with well‐developed papillary cores (**C**), but focally defined invasion is seen (**D** and **E**), together with high‐nuclear grade and frequent mitoses (**F**).

These tumours exhibit EPC‐like low‐power morphology, occasionally with a partial capsule, but are characterised by high‐grade nuclei (Figure 12), high nuclear‐to‐cytoplasmic ratios and elevated mitotic activity, and triple‐negative or HER2‐positive immunoprofiles. In some cases, lymph node metastases with similar morphology are identified.^3^, ^4^, ^26^, ^30^, ^33^, ^34^, ^35^, ^36^ The primary tumours are typically large and often associated with focal conventional stromal invasion. Although they retain an “encysted” papillary pattern like conventional EPC, they have other features more typical of biologically aggressive IBC‐NST, such as circumscribed, expansile borders on low‐power magnification. The clinical and pathological features, in conjunction with the absence of a peripheral MEC layer and frequent evidence of conventional stromal invasion, strongly support the classification of these tumours as invasive.

Cytologically, these tumours resemble high‐grade basal‐like invasive breast cancers.^37^ Key morphological features include increased nuclear‐to‐cytoplasmic ratio, marked nuclear enlargement and pleomorphism, irregular nuclear membranes, hyperchromatic vesicular nuclei with prominent nucleoli, and a mitotic rate of at least mitotic score 2.^34^, ^37^ Geographic necrosis and comedo‐type calcifications can be frequent. These tumours are often hormone receptor‐negative and may be HER2‐positive. Neuroendocrine markers are negative or only focally positive.

As there is morphological overlap between conventional EPC and high‐grade EPC‐like invasive carcinomas, a balanced approach is recommended when reporting and staging these tumours, especially in core needle biopsy, which should prompt surgical excision for thorough histological evaluation, rather than neoadjuvant therapy. Sentinel lymph node biopsy should be advised. Predictive receptor status should be reported on the excision specimen when final classification is completed. Classification and staging of such tumours on the excision specimen should be based on comprehensive examination, particularly of the tumour borders for irregularities, lack or attenuation of peripheral capsule and the presence and extent of stromal invasion, in addition to the presence of other features of invasiveness such as lymphovascular invasion and lymph node metastasis. In tumours with irregular borders and multiple foci of stromal invasion, the whole lesion is considered an invasive disease. However, in tumours with a limited infiltrative nature, the most infiltrative part should be considered as invasive.

While these high‐grade SPC‐like and EPC‐like invasive carcinomas need to be recognised to avoid undertreatment, pathologists should refrain from regarding typical EPC or SPC *in situ* as invasive based solely on higher nuclear grade appearances. A diagnosis of invasive SPC or EPC‐like carcinoma should rely on the constellation of histological and IHC features described above.

##### Differences between EPC and EPC‐like invasive carcinoma

Classical low and intermediate‐nuclear grade, ER‐positive EPCs lacking MECs are not biologically a true *in situ* disease, as demonstrated by multiple reports of metastatic spread in cases without evident conventional stromal invasion.^1^, ^4^, ^38^, ^39^, ^40^, ^41^, ^42^ The surrounding capsule appears to represent a reactive process rather than a thickened native basement membrane.^38^ The consensus in the literature to stage these tumours as *in situ* is primarily driven by their low metastatic potential, which does not typically justify systemic chemotherapy,^40^, ^41^, ^42^, ^43^ together with their indolent histological features and the prevailing clinical practice of staging EPC as DCIS. The same concept is applied to the rounded islands of SPC lacking MECs.

It would not seem entirely logical to consider that a change in grade (e.g., the presence of nuclear grade 3 or receptor status [loss of ER or HER2 positivity]) should alter the classification from *in situ* to invasive disease. Such features are not typical of the progression of an *in situ* lesion to invasive carcinoma but may reflect IBC that has adopted a papillary architecture resembling SPC or EPC. In invasive breast cancer, high‐grade, ER negativity and HER2‐positivity are typically associated with aggressive behaviour. In breast pathology, nuclear grade and receptor status can be used in combination with morphology to classify tumours as benign, atypical, or malignant, with higher grade, ER‐negative, or HER2‐positive phenotypes typically associated with a malignant phenotype and with a more aggressive clinical course and poorer outcomes. For instance, in adenomyoepitheliomas, significant cytological atypia or increased mitotic activity (e.g., >10/10hpf) results in changing the classification from benign/atypical to carcinoma.^9^ Similarly, HER2 positivity (IHC score 3+) supports upgrading an atypical intraductal apocrine epithelial proliferation to a malignant disease (DCIS).^9^

Multiple studies have demonstrated that a large proportion of these high‐nuclear grade EPC‐like lesions exhibit conventional invasive areas in up to 80% of cases.^16^, ^26^, ^30^, ^31^, ^32^, ^34^, ^36^ Other studies have also reported that high‐nuclear grade EPC‐like tumours frequently exhibit lymphoplasmacytic infiltrates, thickened papillae, and larger size (>4 cm), together with a lack of hormone receptor expression, frequent basal‐like immunophenotype, and high Ki‐67 proliferative indices, features favouring invasive disease.^16^, ^26^, ^34^, ^36^ The patients are typically younger than those with conventional EPC, and the lesions frequently present as rapidly enlarging masses or as extensive microcalcifications on screening mammography.^1^, ^16^, ^26^, ^34^, ^36^ In a prior study, we described examples of high‐nuclear grade tumours lacking peripheral MEC, with a high mitotic rate, large size and triple‐negative or HER2‐positive phenotypes, which were misclassified as *in situ* based on the EPC‐ or SPC‐like architecture.^1^ Most of these cases demonstrated focal stromal invasion, lacked a fibrous capsule despite being circumscribed, and had papillary cores which were poorly developed, favouring entrapped stromal tissue within the proliferating tumour cells. We also recently reported two cases of high‐grade papillary carcinomas that mimicked EPC with well‐developed papillary cores and cystic growth on core biopsy, yet showed macrometastases with similar morphology in axillary lymph nodes. These two tumours showed a triple‐negative phenotype and lacked MECs. Thorough examination of the excision specimen revealed irregular borders with scattered foci of definite stromal invasion.^4^

These findings support classifying such tumours as either: (1) invasive lesions mimicking EPC or SPC or (2) EPC or SPC which has acquired additional features indicative of aggressive behaviour, such as an increase in nuclear grade (from 2 to 3), and/or phenotypic drift with ER loss or HER2 positivity. The higher frequency of coexistent areas of conventional invasive carcinoma, together with evidence of metastatic events, warrants classification as invasive disease for management purposes, along with meticulous sampling to identify convincing invasive areas. The same criteria used to stage conventional low‐ and intermediate‐nuclear grade, ER‐positive SPC *in situ* and EPC as *in situ* disease do not apply to these biologically aggressive, high‐nuclear grade invasive tumours that lack MECs. They should be recognised and managed as IBC, reflecting their more aggressive biological behaviour, despite the lack of extensive follow‐up data.^4^, ^16^, ^26^, ^30^, ^36^ Circumscription and papillary architecture do not equate to *in situ* disease.

#### Invasive Papillary Carcinoma NOS (Including Tubulopapillary Pattern)

**Figure 13:**
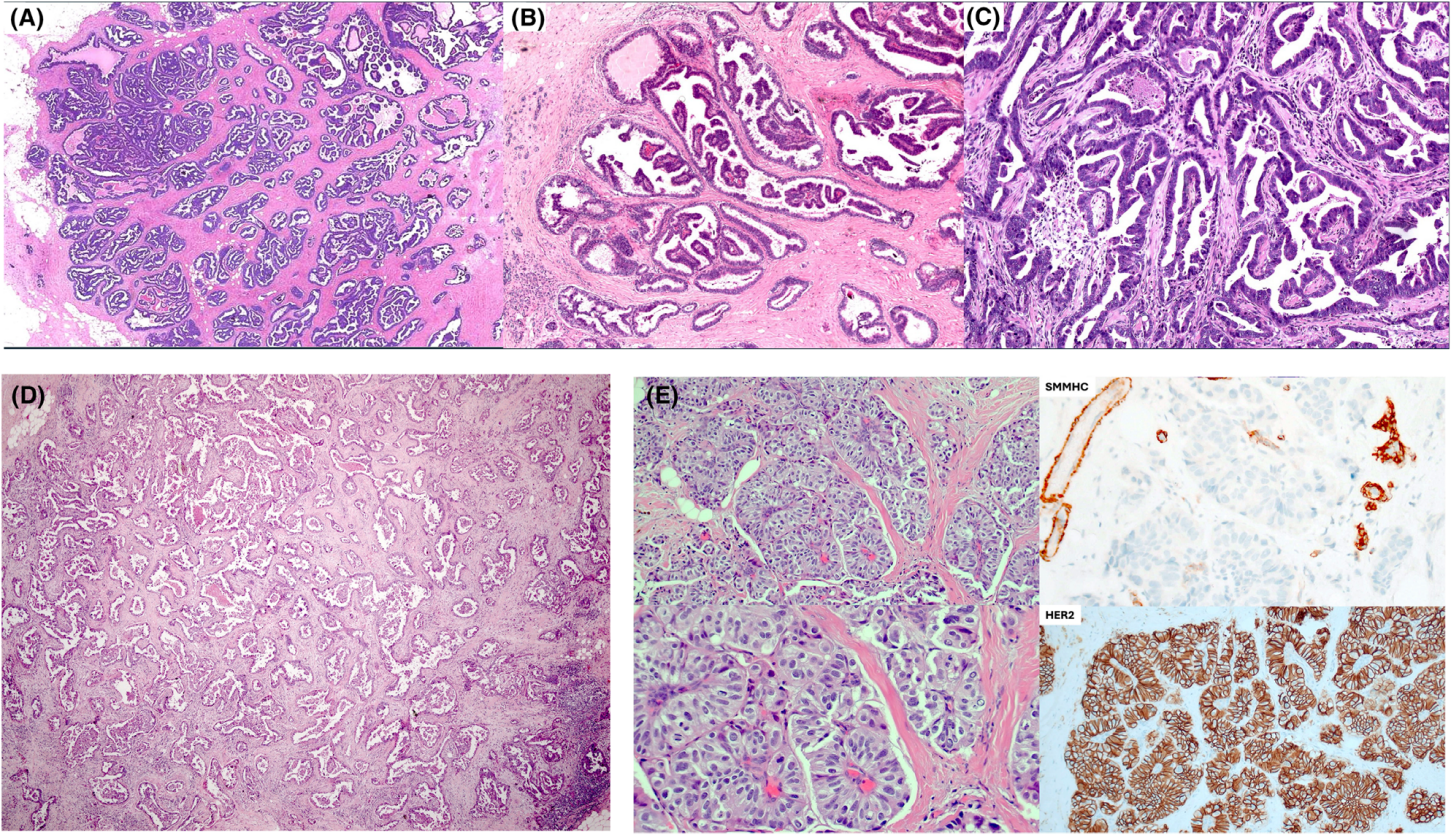
Examples of invasive papillary carcinoma, NOS (IPC), showing small irregular cystic and tubulopapillary structures lacking the defining features of EPC, SPC, or papillary DCIS (**A–D**). The stroma is desmoplastic, with no peripheral capsule or myoepithelial cells at the epithelial–stromal interface. Tubulopapillary structures may exhibit micropapillary projections, as illustrated in (**D**). In (**E**), the IPC demonstrates infiltrative papillary clusters within desmoplastic stroma, lacking tubular or tubulocystic structures. Myoepithelial cells are absent, confirmed by negative smooth muscle myosin heavy chain (SMMHC) expression, and the tumour shows HER2 positivity. These tumours typically score 3 for tubule formation, as tubulopapillary structures are not present in the normal breast. Malignant calcifications may be present, and these tumours can be of high grade. They should be carefully distinguished from metastatic serous papillary carcinoma.

Pure invasive papillary carcinoma of the breast (IPC) is an exceptionally rare type of IBCP defined by the formation of finger‐like fibrovascular projections covered by neoplastic epithelium, which infiltrate desmoplastic or fibrotic stroma in >90% of the tumour (Figure 13). These tumours lack the characteristics of other types of IBCP described above. No data are available on the frequency of IPC, and the reported cases overlap with EPC and SPC, but the term IPC should not be used for non‐papillary carcinomas arising in association with papillary carcinoma *in situ*.

The clinical and macroscopic features are not distinguishable from those of IBC‐NST. Histologically, IPC shows a frankly invasive growth pattern and is composed of papillary formations within microcysts and tubulocystic structures. Individual papillary fronds often blend imperceptibly into invasive nests, cords, or small tubules. MECs are absent at the periphery of the spaces containing the papillary formations and along the papillary stalks.

IPC is not characterised by a circumscribed growth pattern, neuroendocrine differentiation, mucin secretion, or a peripheral fibrous capsule and may show a variable degree of tumour differentiation, including low‐ and high‐nuclear grade. Frequently, IPC is admixed with other invasive carcinoma types, such as invasive micropapillary carcinoma and IBC‐NST. IPC may occasionally mimic papillary DCIS at low‐power examination, with multiple papillary clusters of variable size resembling dilated ducts of papillary DCIS, but IPC is infiltrative and there is no peripheral MEC layer.

A previously characterised tubulopapillary pattern expands the morphologic spectrum of IPC.^44^, ^45^, ^46^ These tumours exhibit a labyrinthine proliferation of variably dilated tubules and cysts, with lumina containing delicate micropapillary or filiform protrusions that recapitulate the papillary cores of classic IPC, but within a tubulocystic architecture (Figure 13). Tubulopapillary architecture is not a feature of EPC or SPC *in situ*, but a feature of invasive carcinoma. In a study of 17 cases of PC, the presence of tubulopapillary features was associated with adverse prognostic features, including significantly higher proliferative activity, nuclear Grade 3, lymphovascular invasion, p53 overexpression, and axillary nodal involvement.^44^, ^45^ High expression of p16 and pathogenic variants of *TP53* in these tumours have been reported.^46^

A recently described serous‐like breast carcinoma has been reported in a study of 15 patients.^47^ All patients were female without a history of gynaecologic malignancy. Histologically, these tumours showed angulated, branched, sometimes anastomosing glands with pseudopapillary luminal projections in a desmoplastic stroma. Most cases were triple‐negative or HER2‐positive, CK5/6‐positive, p16‐positive, Nottingham Grade 2 or 3, and showed frequent *TP53* mutations. It is uncertain how these tumours relate to conventional IPCs.

**Figure 14:**
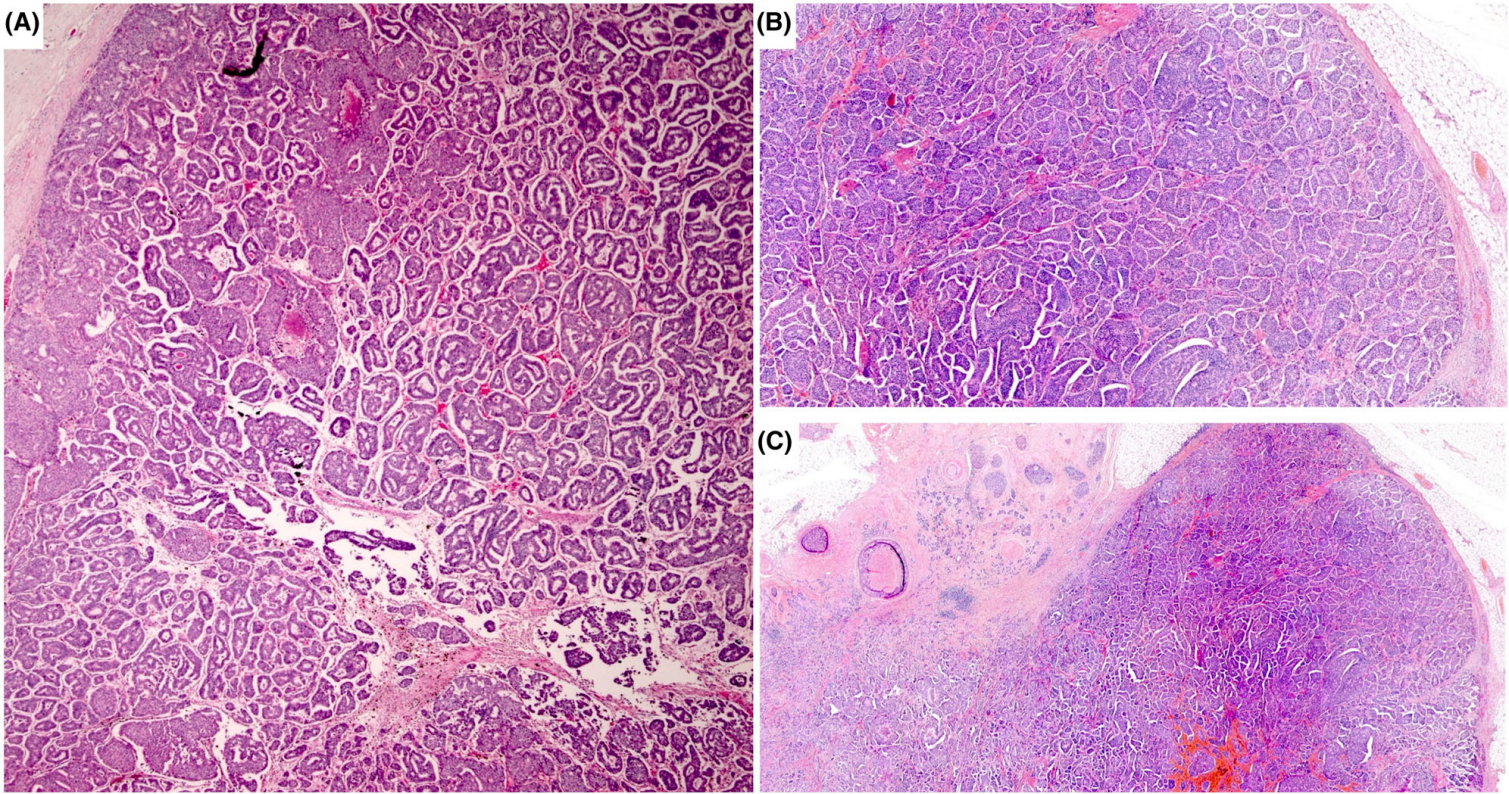
Papillary carcinoma, whether EPC or SPC, associated with an invasive micropapillary component, should be considered as an invasive tumour even if no conventional stromal invasion is seen outside the capsule. (**A**) A higher magnification of invasive micropapillary structures demonstrating the reversal of polarity and clear spaces around the invasive micropapillary structures. (**B**, **C**) Two examples of SPC associated with invasive micropapillary components (low magnification).

IPC should be staged and managed as IBC‐NST. IPC should also be differentiated from the well‐described entity of invasive micropapillary carcinoma, which is characterised by clusters of tumour cells that lack true fibrovascular cores and are surrounded by clear stromal spaces with an “inside‐out” growth pattern (Figure 14).

##### Diagnostic considerations and differential diagnosis

**Figure 15:**
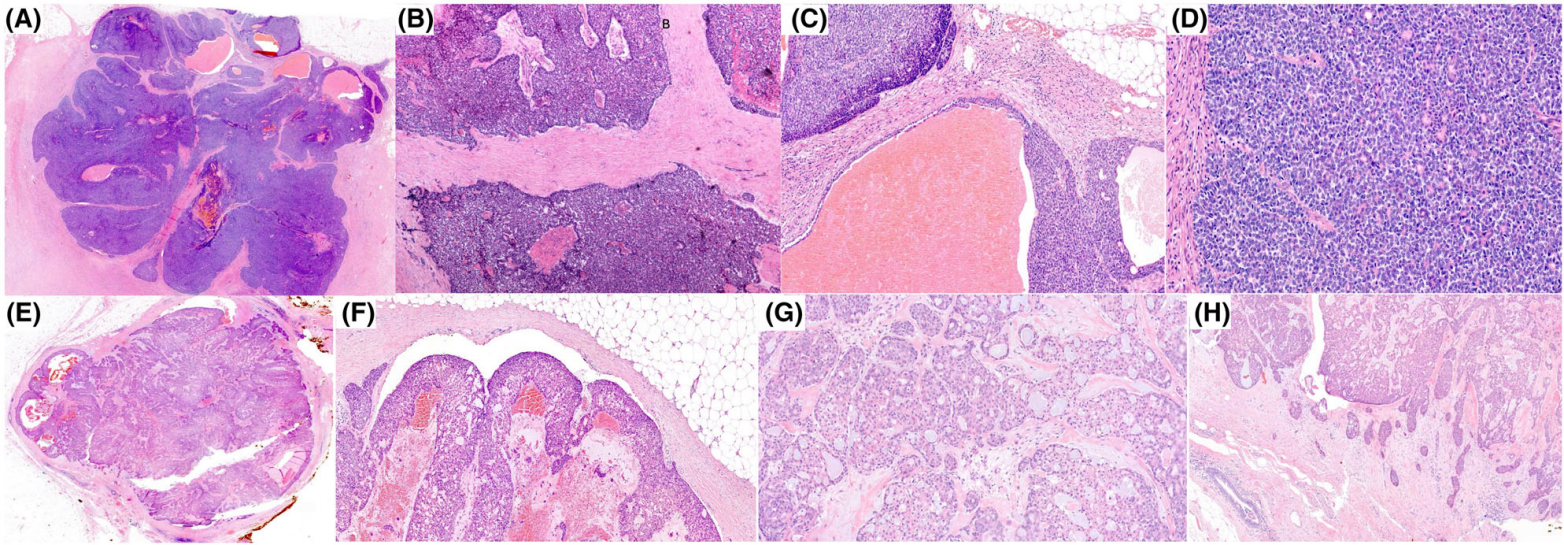
Examples of invasive carcinoma with papillary architecture, including adenoid cystic carcinoma (**A**–**D**) and secretory carcinoma (**E**–**H**), at different magnifications. The diagnosis of both tumours was confirmed by immunohistochemistry (data not shown).

Accurate diagnosis of IBCP subtypes requires meticulous histological evaluation, supported by immunohistochemistry. However, the diagnosis is often complicated by morphological and immunophenotypic overlap with benign papillary lesions (papillomas with or without atypia or carcinoma *in situ*) and papillary DCIS, both of which retain a peripheral MEC layer. Classical EPC, which lacks MECs, and SPC, which may be devoid of MECs, are classified as *in situ* or invasive based on the presence or absence of unequivocal stromal invasion. Several other rare breast and non‐breast tumours can mimic IBCP morphologically or immunohistochemically (see below). Accurate distinction is crucial as these entities differ significantly in terms of prognosis, therapeutic implications, and site of origin. In such cases, a comprehensive assessment incorporating imaging findings, clinical context, cytological features, and immunohistochemistry is critical for accurate classification. Adherence to standardised diagnostic criteria improves diagnostic consistency and guides appropriate clinical management. IBCP should also be differentiated from the following tumours that may exhibit papillary or solid papillary architecture:

*Secretory carcinoma*: This tumour typically affects younger individuals and often displays a variety of morphologies, including papillary (Figure 15), microcystic, solid, and tubular patterns with abundant eosinophilic or vacuolated (“bubbly”) cytoplasm. Unlike IBCP, secretory carcinoma is strongly S100‐positive and expresses mammaglobin and GCDFP‐15, while it is typically negative or only weakly positive for oestrogen receptor (ER). The ETV6–NTRK3 gene fusion is characteristic and can be confirmed by molecular testing or surrogate NTRK immunohistochemistry.

*ILC with papillary features*: Rare cases of ILC may exhibit focal papillary architecture, but they typically display dyscohesive single‐cell growth, targetoid or “Indian file” patterns, and a lack of tubule formation.^12^ The hallmark is complete loss of E‐cadherin expression, which distinguishes ILC from IPC. IBCP retains E‐cadherin and typically lacks the classic lobular growth pattern.

*Adenoid cystic carcinoma*: Adenoid cystic carcinoma (AdCC) may show cribriform and pseudopapillary architecture with dual epithelial and MEC populations (Figure 15). The presence of true basement membrane‐like material and hyaline globules is characteristic. IHC reveals dual cell populations with positivity for CK7 in epithelial cells and p63/SMMHC in MECs. AdCC is usually triple‐negative (ER/PR/HER2) and expresses CD117 (c‐kit), aiding in distinction from IBCP, which typically shows luminal marker expression and lacks c‐kit.

*Adenomyoepithelioma with papillary architecture*: This biphasic tumour consists of epithelial and myoepithelial cell components and can mimic papillary neoplasms. It often shows a solid or papillary architecture in association with the prominent myoepithelial differentiation. MEC markers are positive, contrasting with IBCP, where these markers are absent or limited. In benign adenomyoepithelioma (AME), both cell populations are generally bland, and cytological atypia is minimal; however, malignant AME shows malignant cytological features, and the distinction between the two cell populations becomes less distinct. In such cases, identification of the biphasic cytological features, ER negativity, and MEC marker expression, at least focally, supports their diagnosis as malignant AME rather than EPC or SPC.

*Tall cell carcinoma with reversed polarity*: IBCP lacks the distinctive nuclear and architectural features of TCCRP.^9^ TCCRP is a rare low‐grade triple‐negative IBC characterised by solid and papillary architecture with reversed nuclear polarity (nuclei aligned away from the basement membrane). TCCRP is composed of columnar cells arranged in nests, papillae, and follicle‐like structures. Nuclear grooves are typically observed, and ground glass‐appearing nuclei, nuclear pseudo‐inclusions, and psammoma bodies may be present, mimicking the tall cell variant of papillary thyroid carcinoma.^48^, ^49^ Although most of these features can be seen focally in other papillary lesions, the constellation of all distinguishes TCCRP. TCCRP typically shows strong CK5/6 and CK7 expression and lacks hormone receptor expression. Notably, these tumours are characterised by specific mutations involving *IDH2* and *PIK3CA* genes and are positive for monoclonal antibodies (11C8B1) directed against IDH2 R172.^50^ TTF1 and thyroglobulin are negative, excluding metastatic thyroid carcinoma.

*Mucinous cystadenocarcinoma*: A rare type of breast carcinoma that may mimic mucinous cystadenocarcinoma of the ovary, pancreas, or appendix, as well as cystic hypersecretory carcinoma of the breast.^9^ The tumour shows cystic spaces lined by bland, tall columnar cells with abundant intracytoplasmic and extracellular mucin, together with cellular stratification, tufting, and papillary formation. Grossly, the tumour is often multicystic and may resemble cystic papillary carcinoma.

*Metastatic papillary carcinomas*: IBCP should also be differentiated from metastatic papillary carcinomas from other organs, which may present as multiple but can also be solitary masses. Key immunohistochemical distinguishing antibodies are ER, PR, HER2, GATA3, GCDFP‐15, mammaglobin, E‐cadherin, TTF1, thyroglobulin, WT1, PAX8, and NTRK. The most common metastatic papillary tumours to the breast are listed below.

1. Papillary thyroid carcinoma: expresses TTF1 and thyroglobulin; negative for ER/PR and GATA3.
2. Uterine, ovarian, and tubal papillary serous carcinoma: express IHC markers PAX8, P53, WT1 (less frequent in uterine serous carcinoma), and p16; may express CA‐125; negative for GATA3, SOX10, GCDFP‐15, and mammaglobin. ER can be positive but is typically weak and focal.
3. Lung adenocarcinoma: expresses TTF1 and napsin A; negative for breast markers (ER, PR, GATA3, SOX10, mammaglobin). Rare GATA3‐positive lung adenocarcinomas have been reported.^51^

Another rare tumour that may show papillary architecture and should be considered is metastatic papillary renal cell carcinoma.^3^ We have also seen a case of metastatic medullary carcinoma of the thyroid to the breast that presented as a solitary metastasis and was initially misclassified as a primary IPC of the breast. The lesion had a papillary architecture, and IHC confirmed the diagnosis of medullary thyroid carcinoma.

### Genes and Pathways in Papillary Morphogenesis

Genetic and molecular studies implicate several pathways in papillary morphogenesis, including the hepatocyte growth factor receptor *c‐Met*, *RET/PTC*, *α3β1* integrin, Sonic Hedgehog (*Shh*), and Bone Morphogenetic Protein (*BMP*).^52^, ^53^, ^54^, ^55^, ^56^ (Table 2). However, papillary morphogenesis is not a feature of normal breast development, and its role in neoplasia remains incompletely understood. Benign intraductal papillary lesions of the breast are monoclonal in origin.^60^ Studies have demonstrated activating mutations in the *PIK3CA/AKT1* pathway, as well as frequent loss of heterozygosity (LOH).^57^, ^58^ In a small series, LOH on chromosome 16p13 was observed in 60% of intraductal papillomas (IDPs) with florid usual ductal hyperplasia (UDH), with additional LOH at 16q23 in papillary carcinomas.^61^ Other chromosomal aberrations, including alterations in chromosomes 3, 7, 17, and X, have been reported in 15%–21% of papillary carcinomas but not in IDPs.^62^

**Table 2:** Genes and pathways in papillary morphogenesis^52^, ^53^, ^54^, ^55^, ^56^, ^57^, ^58^, ^59^

Gene/pathway	Role	Relevance
*HGF/c‐MET*	Regulates epithelial proliferation, motility, and branching	Implicated in normal ductal branching and tumour progression with papillary morphology
*RET/PTC*	Involved in epithelial–mesenchymal interaction and branching morphogenesis	Expressed in papillary thyroid and breast tumours; linked to papillary architecture
*α3β1* Integrin	Mediates cell–extracellular matrix interactions and branching	Drives stromal–epithelial signalling essential for morphogenesis
Sonic Hedgehog (*Shh*)	Regulates ductal patterning and branching during development	Aberrant activation linked to neoplastic growth with papillary features
Bone Morphogenetic Proteins (*BMPs*)	Controls morphogenesis via SMAD signalling; modulates the stem cell niche	Dysregulation contributes to oncogenesis and architectural complexity
*PIK3CA/AKT1* Pathway	Promotes cell survival and growth	Frequently mutated in benign and malignant papillary breast lesions
*TP53*	Regulates apoptosis and genomic integrity	Relevant in progression to invasive disease
*GATA3*	Critical for luminal differentiation	Expressed in papillary tumours; maintains epithelial identity
*FOXA1*	Regulates ER activity and ductal patterning	Maintains papillary phenotype with GATA3
*ERBB2* (HER2)	Involved in cell growth and polarity	Overexpressed in SPC and EPC with apocrine features
MUC1	Regulates apical‐basal polarity	Redistribution in invasive micropapillary carcinoma; preserved in true papillary lesions
*VEGF*	Promotes angiogenesis	Supports vessels in fibrovascular cores of papillary structures

A study of 89 papillary lesions found mutations in 62% of cases, primarily involving *AKT1* and *PIK3CA*.^57^ Mutation patterns varied across subtypes: IDP without atypia: *AKT1* (54%) and *PIK3CA* (21%); IDP with UDH: *PIK3CA* (42%) and *AKT1* (15%); IDP with ADH: *PIK3CA* (45%), *AKT1* (27%), and *NRAS* (9%); and in papillomas with DCIS, 3 of 7 cases harboured *AKT1* mutations.^57^

Genomic profiling of papillary carcinoma (including EPC and SPC) showed a similar mutation profile to grade‐ and ER‐matched infiltrating ductal carcinoma but with fewer copy number aberrations. EPC exhibited the fewest alterations, followed by DCIS and then invasive carcinoma, suggesting that EPC is genetically closer to in situ disease.^63^ Compared with IBC‐NST, papillary carcinomas exhibited downregulation of genes associated with proliferation, motility, and cellular assembly, as well as upregulation of genes related to homeostasis and angiogenesis, indicating a less aggressive phenotype.

### Prognosis and Clinical Management

There is limited data on the behaviour of many of these individual tumour types, but given the relatively favourable prognosis often associated with circumscribed papillary architecture,^5^, ^7^, ^8^ a pragmatic approach should be taken when evaluating the invasive component, especially in large tumours, to avoid overtreatment. In our practice, only definite invasive foci are considered representative for staging and receptor analysis. The entire circumscribed component is included when measuring overall tumour size (Figure 4), while still staging the tumour as invasive to reflect its metastatic potential. At present, prognostic and predictive multigene assays (such as Oncotype DX and MammaPrint) have not been validated in the rare subtypes of IBC, such as IBCP. Given their rarity and biological heterogeneity, current evidence does not support routine use of these tests in this group. Treatment decisions should instead be guided by established morphological features, histological grade, ER/PR/HER2 status, and other conventional clinicopathological parameters, which remain the most reliable approach in the absence of subtype‐specific genomic validation.

Overall, 5‐year disease‐specific survival of IBCP exceeds 90%, and axillary nodal metastases are uncommon. Accordingly, treatment follows standard breast‐conservation or mastectomy principles with sentinel‐node evaluation; adjuvant endocrine therapy is recommended for hormone receptor‐positive cases, and chemotherapy is reserved for cases with high‐risk features (e.g., large size, high‐grade morphology, triple‐negative or HER2‐positive phenotype, and nodal involvement).^5^, ^7^, ^8^

## Conclusion

IBCP encompasses diverse entities with varying diagnostic challenges and prognostic implications. Awareness of these entities allows accurate classification by pathologists to guide optimal clinical management. Continued research into their molecular profiles may further refine diagnostic criteria and therapeutic strategies for these heterogeneous tumours.

## Author contributions

ER drafted the manuscript and approved the final version. PHT and WR amended the manuscript and approved the submitted version.

## Funding statement

The authors have nothing to report.

## Conflict of interest statement

No conflict of interest to declare.

## Data Availability

Data sharing not applicable to this article as no datasets were generated or analysed during the current study.
